# TAGLN2 induces resistance signature ISGs by activating AKT-YBX1 signal with dual pathways and mediates the IFN-related DNA damage resistance in gastric cancer

**DOI:** 10.1038/s41419-024-07000-1

**Published:** 2024-08-21

**Authors:** Huiqin Zhuo, Jingjing Hou, Zhijun Hong, Shuqi Yu, Huifang Peng, Lihua Zhang, Wen Xie, Xuehui Hong

**Affiliations:** 1 Department of Gastrointestinal Surgery, Zhongshan Hospital of Xiamen University, School of Medicine, Xiamen University, Xiamen, China; 2 Xiamen Municipal Key Laboratory of Gastrointestinal Oncology, Xiamen, China; 3 Department of Pathology, Zhongshan Hospital of Xiamen University, School of Medicine, Xiamen University, Xiamen, China; 4 Department of Endocrinology, The First Affiliated Hospital and College of Clinical Medicine of Henan University of Science and Technology, Luoyang, China

**Keywords:** Cancer therapy, Predictive markers

## Abstract

Recently, various cancer types have been identified to express a distinct subset of Interferon-stimulated genes (ISGs) that mediate therapy resistance. The mechanism through which cancer cells maintain prolonged Interferon stimulation effects to coordinate resistance remains unclear. Our research demonstrated that aberrant upregulation of TAGLN2 is associated with gastric cancer progression, and inhibiting its expression renders gastric cancer cells more susceptible to chemotherapy and radiation. We uncovered a novel role for TAGLN2 in the upregulation of resistance signature ISGs by enhancing YBX1-associated ssDNA aggregation and cGAS-STING pathway activation. TAGLN2 modulates YBX1 by recruiting c-Myc and SOX9 to *YBX1* promoter region and directly interacting with AKT-YBX1, thereby enhancing YBX1 phosphorylation and nuclear translocation. Significantly, targeted downregulation of key proteins, inhibition of the TAGLN2-YBX1-AKT interaction (using Fisetin or MK2206) or disruption of the cGAS-STING pathway substantially reduced ssDNA accumulation, subsequent ISGs upregulation, and therapy resistance. The combination of Cisplatin with MK2206 displayed a synergistic effect in the higher *TAGLN2*-expressing xenograft tumors. Clinical analysis indicated that a derived nine-gene set effectively predicts therapeutic sensitivity and long-term prognosis in gastric cancer patients. These findings suggest that TAGLN2, YBX1 and induced ISGs are novel predictive markers for clinical outcomes, and targeting this axis is an attractive therapeutic sensitization strategy.

## Introduction

Interferon (IFN) signaling plays a pivotal role in the efficacy of conventional cancer therapeutic strategies, attributed to its critical functions in early antigen recognition and its capacity to bridge innate sensing with the adaptive immune response [1–3]. Therapy employing IFNs alone, or in combination with chemo-, radio-, or immunotherapy, has shown substantial effectiveness in treating a variety of cancer types [4, 5]. On this basis, type I or II IFN-stimulated genes (ISGs) have emerged as prognostic biomarkers for cancer therapeutic strategies. However, recent contradictory observations concerning IFNs in cancer therapy have emerged [6]. Radiation-induced activation of the STING/type I IFN pathway enhances tumor-suppressive inflammation by recruiting myeloid cells through the CCR2 pathway, and treatments targeting CCR2 have mitigated the immunosuppressive effects of radiotherapy and STING agonist strategies [7]. Cancer cells that acquire epigenetic features of inflammatory memory, promoted by prolonged IFN signaling, perpetuate immune dysfunction [8]. Accumulating evidence suggests that the expression of a subset of resistance signature ISGs leads to an IFN-related DNA damage resistance signature (IRDS) [9], marking a significant advancement in understanding cancer resistance mechanisms. In one study, a seven-gene-pair set served as both a clinical classifier for IRDS status and a predictive marker for chemoradiotherapy response [10], highlighting the therapeutic potential of targeting these ISGs to overcome resistance to cancer treatment [10, 11]. Moreover, various untreated human primary cancer types overexpress IRDS genes, suggesting a common and inherent resistance mechanism and underscoring the potential of modulating this response across different cancer types.

Further investigation has indicated that IRDS genes are primarily regulated by IFN signaling in cancer cells, distinguishing their expression from the IFNG GSEA Hallmark gene set, which is predominantly expressed by intratumoral immune cells [12]. Selective induction of IRDS genes can be triggered through elevated expression of specific factors or chronic exposure to low levels of IFN-β, without affecting other antiproliferative or proapoptotic ISGs [9, 13]. Additionally, chronic exposure to DNA-damaging agents or defects in cellular DNases, or the presence of chromosomal instability, may lead to sustained low levels of IFN activation following DNA damage [9, 13]. Erdal et al. demonstrated that DNA-damaging agents induced higher Bloom syndrome (BLM) helicase and exonuclease 1 (EXO1) expression levels for DNA end resection but lower Trex1 expression levels for cytosolic DNA degradation, which led to the release of single-stranded DNA (ssDNA) fragments from the cell nucleus into the cytosol [14]. Upregulated expression of *DDX60, STAT1, OAS1, IFI6,* and *IFI27* genes are common features in tamoxifen- and radiotherapy-resistant breast cancer and are induced by cytosolic ssDNA aggregation in a dose- and time-dependent manner [15]. Both self and nonself cytosolic DNA molecules (ssDNA and dsDNA) can trigger cyclic guanosine monophosphate (GMP)-adenosine monophosphate (AMP) synthase (cGAS)-STING pathway signaling, which upregulates IRDS gene expression. However, more studies are needed to further understand the upstream cause of aberrant self-cytosolic DNA aggregation in various untreated primary cancer types with high resistant signature ISG gene expression and an IRDS. IRDS genes mediate resistance to DNA damage and are a predictive marker for chemoradio- and immunotherapy outcomes.

TAGLN2, a member of the actin-binding protein family, although not fully understood, plays a role in cancer by being upregulated in response to external inflammatory signals [16, 17]. Its upregulation has been associated with tumorigenesis, cancer progression, and therapy resistance, suggesting its potential as an oncogenic factor [18]. Studies have identified TAGLN2 among other prognostic hub genes as predictors of prognosis in certain cancer types, indicating a potential relationship between TAGLN2 and IFN-induced genes [19].

This study demonstrated that TAGLN2 expression is upregulated in the early stages of gastric cancer and increases with tumor progression, and suggested the involvement of dual regulatory pathways in upregulating resistance signature ISGs, with cytosolic ssDNA aggregation playing a crucial intermediary role. Identifying key molecules in this signaling axis as targets may offer new avenues to reduce resistance to DNA-damaging therapies and serve as predictive markers for therapy response in gastric cancer.

## Results

### Aberrantly upregulated expression of TAGLN2 results in the induction of ISGs

**Fig. 1:**
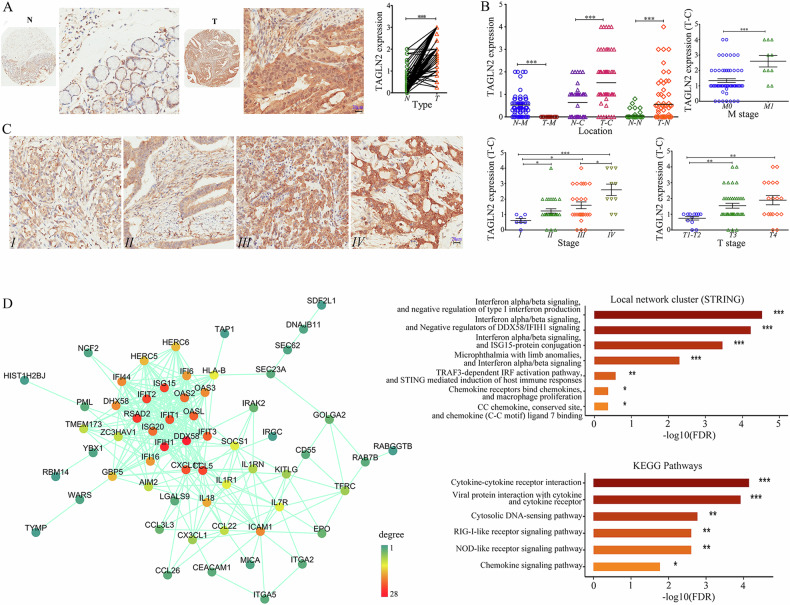
Aberrantly upregulated expression of TAGLN2 results in the induction of ISGs. **A** Aberrantly upregulated TAGLN2 protein expression was assessed by IHC analysis in gastric cancer (GC) tissue microarray (TMA) chips containing 75 tumor tissues and their paired normal counterparts. **B** Cellular TAGLN2 expression in tumor tissues vs. paired normal counterparts was evaluated in the membrane, cytoplasm and nucleus. Distant metastases (M), T stage and overall cancer stage (Stage I~IV) were significantly correlated with TAGLN2 expression in the cytoplasm. **C** Representative IHC images show that abnormal TAGLN2 expression level increases as GC progresses (Stage I~IV). The results were obtained by multiplying the PP by the IS (immunoreactive score = PP × IS). *P < 0.05, **P < 0.01, ***P < 0.001. **D** The combined analysis of the RNA‒Seq and iTRAQ-2DLC‒MS/MS analyses showed that 92 upregulated TAGLN2-associated genes constituted a tight protein‒protein interaction network and KEGG analysis showed that these genes were particularly enriched in 6 important signaling pathways. Proteins in the network were colored based on the degree.

To elucidate the clinical relevance of TAGLN2 in gastric cancer (GC), tissue microarrays comprising 75 tumor tissues and their paired normal counterparts from patients with comprehensive clinical data underwent immunohistochemistry (IHC) analysis. Notably, TAGLN2 staining in tumor tissues was significantly pronounced (score: 2.109 ± 0.098) compared to normal tissues (score: 0.656 ± 0.071; *P* < 0.0001, Fig. 1A). In 85.3% of GC patients, TAGLN2 expression in tumor tissue markedly exceeded that in paired normal counterparts, and high TAGLN2 expression (score ≥ 2) observed more frequently in tumor tissues (68% vs. 8%). Intriguingly, cellular TAGLN2 expression was broadly distributed, being localized at the membrane, cytoplasm, and/or nucleus, which was further underscored (Fig. 1B). The tumor tissues predominantly exhibited negligible TAGLN2 expression at the cancer cell membrane, yet approximately 81% of adjacent normal tissues displayed positive TAGLN2 staining at the membrane. The presence of TAGLN2 in the cytoplasm or nucleus was significantly greater in tumor tissues than in adjacent normal tissues (*P* < 0.0001). Remarkably, about 48% of tumor tissues showed positive TAGLN2 staining in the nucleus (22.5% of which had a score ≥1), a figure substantially higher than that of adjacent normal tissues (9.3%). Distant metastases (M), T stage, and overall cancer stage (Stage I~IV) exhibited a significant association with TAGLN2 expression in the cytoplasm (Supplementary Table 1). Representative IHC images illustrate the aberrant expression of TAGLN2 at the early stage of GC (Stage I) and a progressive increase with tumor advancement (Fig. 1C).

TAGLN2 expression was assessed in 13 GC cell lines and the human gastric mucosal cell line GES-1. Expression levels of TAGLN2 were found to be very low in HGC-27, MKN-45, MKN-28, SNU-5 and SNU-1 cell lines; moderate in HS-746T, MGC-803, AGS and NCI-N87 cell lines; and high in the BGC-823, SGC-7901, KATOIII and SNU-16 cell lines (Supplementary Fig. 1A). To explore the modulation of gene expression due to aberrant TAGLN2 expression, RNA-Seq and iTRAQ-2DLC‒MS/MS analyses were conducted to examine transcriptomic and proteomic alterations in BGC-823 cells. A total of 11,885 unique peptides corresponding to 2778 proteins with 99% confidence were identified. Relative to the control group, the *TAGLN2* overexpression group exhibited 62 differentially expressed proteins (DEPs, 46 up- and 16 downregulated proteins). Among these DEPs, numerous upregulated proteins, including IFIT1, ISG15, IFIT2, IFIT3, OAS3, IFI16, WARS, and TAP1, were identified as products of IFN-stimulated genes. Additionally, several proteins closely associated with the IFN signaling pathway were also significantly upregulated, such as ZC3HAV1, MVP, HLA-B, and PML. The protein expression profile in BGC-823, HGC-27, and SGC7901 cells with aberrant TAGLN2 expression was validated via Western blot (Supplementary Fig. 1B). The protein‒protein interaction network underscored the intimate association among these upregulated proteins (Supplementary Fig. 1C), which were primarily implicated in immune responses, the defense response to viruses, and the type I IFN signaling pathway, particularly involving DDX58/IFIH1 signaling or ISG15-protein conjugation.

Based on RNA-Seq data, 72 common differentially expressed genes (DEGs) were pinpointed in samples with either *TAGLN2* overexpression or knockdown. A combined analysis of the transcriptome and proteome revealed that 92 TAGLN2-associated upregulated proteins or genes (Supplementary Table 2) formed a cohesive protein‒protein interaction network (Fig. 1D). These proteins and genes were significantly enriched in cytokine‒cytokine receptor interactions, the cytosolic DNA-sensing pathway, the NOD-like receptor signaling pathway, and the RIG-I-like receptor signaling pathway. Notably, the upregulated genes *TMEM173 (STING1), AIM2, CCL5, IL18, DDX58 (RIG-I-Like Receptor 1), DHX58 (RIG-I-Like Receptor 3), IFIH1*, and *ISG15* were engaged in the cytosolic DNA-sensing or RIG-I-like receptor signaling pathway (cytosolic RNA-sensing). Both pathways involve cytosolic pattern recognition receptors (PRRs), which trigger the production of type I IFN and instigate a rapid and robust innate immune response.

### TAGLN2 mediates therapy resistance and ISGs upregulation by inducing the accumulation of cytosolic ssDNA

**Fig. 2:**
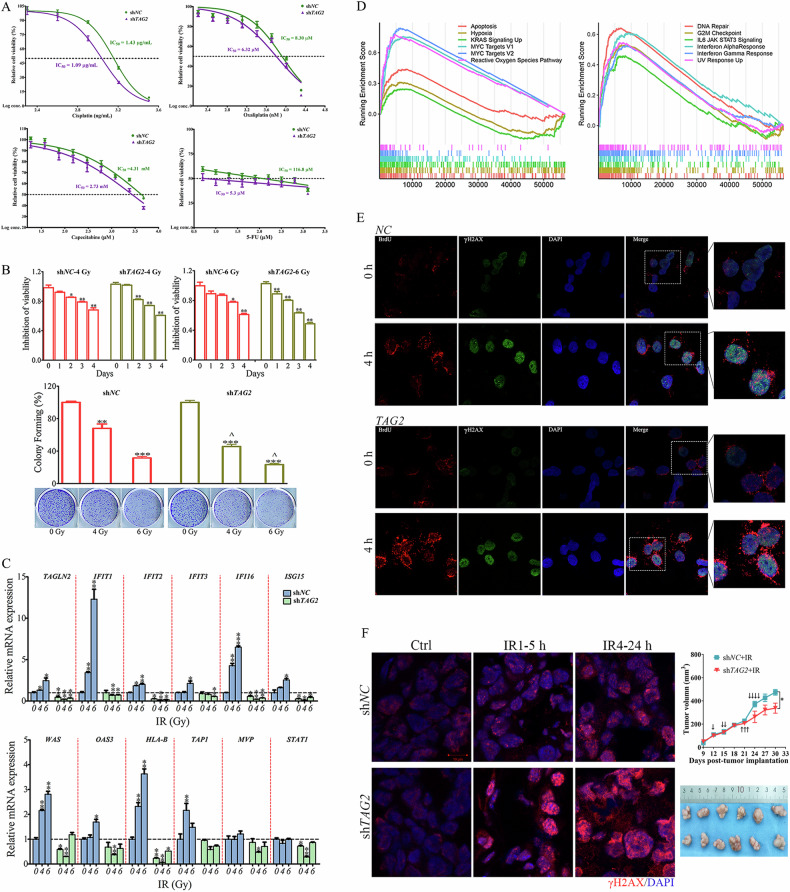
Upregulated TAGLN2 expression induces chemo- and radiotherapy resistance by cytosolic ssDNA mediated ISG upregulation in GC cell lines. **A** The IC_50_ of Cisplatin, Oxaliplatin, Capecitabine and 5-FU were calculated in BGC-823 cells with TAGLN2 downregulation using Cell Counting Kit-8. TAGLN2 was abbreviated as TAG2. **B** The effect of X-ray irradiation on cell viability and clonogenic survival in both TAGLN2 downregulation (sh*TAG2*) and control (sh*NC*) BGC-823 cells irradiated at doses of 0, 4, and 6 Gy X-rays. *P < 0.05, **P < 0.01, ***P < 0.001, treatment vs. before treatment. ^*P* < 0.05, ^^*P* < 0.01, ^^^*P* < 0.001, sh*TAG2* vs. sh*NC* at 4 Gy or 6 Gy, respectively. **C** Relative mRNA levels of *TAGLN2* and the panel of IFN-related genes prior to and after 0, 4, and 6 Gy X-ray treatment were evaluated by quantitative RT‒PCR analysis in both sh*TAG2* and sh*NC* BGC-823 cells. **D** Hallmark pathway analysis by gene set enrichment analysis (GSEA) using a stomach adenocarcinoma (STAD) dataset from The Cancer Genome Atlas (TCGA). **E** Overexpression of TAGLN2 induces the accumulation of cytosolic ssDNA as evaluated by BrdU-γH2AX double labeling. Cells were stained for the primary BrdU antibody (red) and phospho-histone H2AX (green). **F** Representative images of tumors in different groups harvested at 5 h after one dose radiation (IR1-5 h) or 24 h after four doses of radiation (IR4-24 h) were analyzed by immunofluorescence with the antibody against phospho-histone H2AX (red). Slides were mounted with VectaShield antifade mounting medium with DAPI (blue). Images were taken using a 63× objective on a Zeiss LSM780 confocal microscope.

The IC_50_ values of Cisplatin, Oxaliplatin, Capecitabine, and 5-FU treatment all decreased after *TAGLN2* knockdown. Specifically, the IC_50_ value of 5-FU treatment decreased up to 22-fold with TAGLN2 downregulation (Fig. 2A). Conversely, in HGC-27 cells overexpressing TAGLN2 and treated with multiple anti-tumor drugs, an opposite trend was observed (Supplementary Fig. 2A, C). Knocking down *TAGLN2* in the drug-resistant cell line SGC-7901/5-FU significantly restored the cell’s response to 5-FU, with the IC_50_ reduced from 57.94 to 28.29 μM (Supplementary Fig. 2B). X-ray irradiation (IR) on cell viability and clonogenic survival assays confirmed radiosensitization by *TAGLN2* knockdown (Fig. 2B). *TAGLN2* overexpression significantly prevented the PARP cleavage in cells induced by Dox, VP16, and IFNγ (Supplementary Fig. 2D), establishing that TAGLN2 enhances tumor chemoradio resistance in vitro.

RT‒qPCR analysis confirmed the activation of *TAGLN2* and a panel of TAGLN2-induced ISGs *(IFIT1, IFIT2, IFIT3, IFI16, ISG15, HLA-B, OAS3, WARS*, and *TAP1*), with the exception of *MVP* and *STAT1*, after IR treatment in a dose-dependent manner in control cells (Fig. 2C). A significant increase in *IFIT1* expression was observed within 48 h of 6 Gy of IR treatment, and significant induction could also be observed at a lower dose of 4 Gy. However, depletion of *TAGLN2* significantly inhibited the expression levels of the gene panel prior to and after IR treatment, disrupting the dose-dependent increase. Cisplatin treatment resulted in a similar mRNA expression upregulation in the ISGs panel in BGC-823 control cells and inhibition in *TAGLN2*-depleted cells. An opposite trend was observed in HGC-27 cells overexpressing TAGLN2 (Supplementary Fig. 2E), confirming the role of TAGLN2 in the activation of ISGs upon chemotherapeutic or irradiation treatment and implying a mechanism of resistance regulation.

Gene set enrichment analysis (GSEA) Hallmark pathway analysis using a STAD dataset from TCGA revealed that *TAGLN2* is involved in many signaling pathways associated with cancer, including apoptosis, hypoxia, MYC targets, KRAS signaling, and the reactive oxygen species pathway. Importantly, upregulated TAGLN2 was significantly enriched in IFN alpha/gamma response, DNA repair, G2/M checkpoint, and IL6 JAK STAT3 signaling, consistent with our omics results, suggesting that TAGLN2 plays a critical linkage role between DNA repair and IFN signaling activation (Fig. 2D).

The cellular DNA was not denatured, therefore, any BrdU foci observed corresponded to labeled ssDNA. Cisplatin treatment significantly increased the fluorescent signal of γH2AX and BrdU foci in the nucleus at 2 h in HGC-27 cells, and an accumulation of cytosolic BrdU foci was also evident (Fig. 2E and Supplementary Fig. 3A). The signal of cytosolic BrdU foci was most significant at 4 h and persisted for more than 8 h. Strikingly, compared with the control, overexpression of *TAGLN2* substantially induced both nuclear and cytosolic ssDNA accumulation prior to and after Cisplatin treatment. The red fluorescent signal of cytosolic BrdU foci in the *TAGLN2* overexpression group was much stronger than that in the control group at both 4 h and 8 h after Cisplatin treatment. However, γH2AX, which represents the level of double-strand breaks, exhibited a decreased signal in the nucleus both prior to and after Cisplatin treatment in *TAGLN2*-overexpressing cells compared to control cells. Depletion of *TAGLN2* markedly reduced cytosolic ssDNA accumulation and increased γH2AX signal in the nucleus (Supplementary Fig. 3B).

The xenograft tumors treated with IR in vivo were analyzed by immunofluorescence, and the γH2AX foci in the tumors of the sh*TAGLN2* group were significantly more accumulated than that in the sh*NC* group both 5 h after a radiation dose of 250 cGy or 24 h after four radiation doses exposure (Fig. 2F). Tumor growth was modestly slowed by the *TAGLN2* knockdown. Together, these results demonstrated that TAGLN2 plays a key role in DNA repair, generating cytosolic ssDNA fragments, and then activating ISGs.

### YBX1 plays a pivotal role in TAGLN2-induced ISGs upregulation and is transcriptionally regulated by TAGLN2

To pinpoint the origins of cytosolic ssDNA fragments, Coimmunoprecipitation (Co-IP) followed by LC‒MS/MS was utilized to identify TAGLN2-binding candidates. The top five identified candidate proteins were YBX1, HIST1H1D, Actin, Hsp90AB1, and ANXA2 (Supplementary Table 3). Among these, YBX1, also known as nuclease-sensitive element-binding protein 1, scored the highest at 233 and is involved in numerous DNA repair pathways. Additionally, omics analysis (Fig. 1D) revealed that YBX1 was significantly increased with TAGLN2 overexpression, and acted as a potential upstream factor of IFN signaling activation or the cytosolic DNA-sensing pathway. Consequently, YBX1 was deemed the most intriguing candidate target of TAGLN2, potentially regulated at both the mRNA and protein levels.

**Fig. 3:**
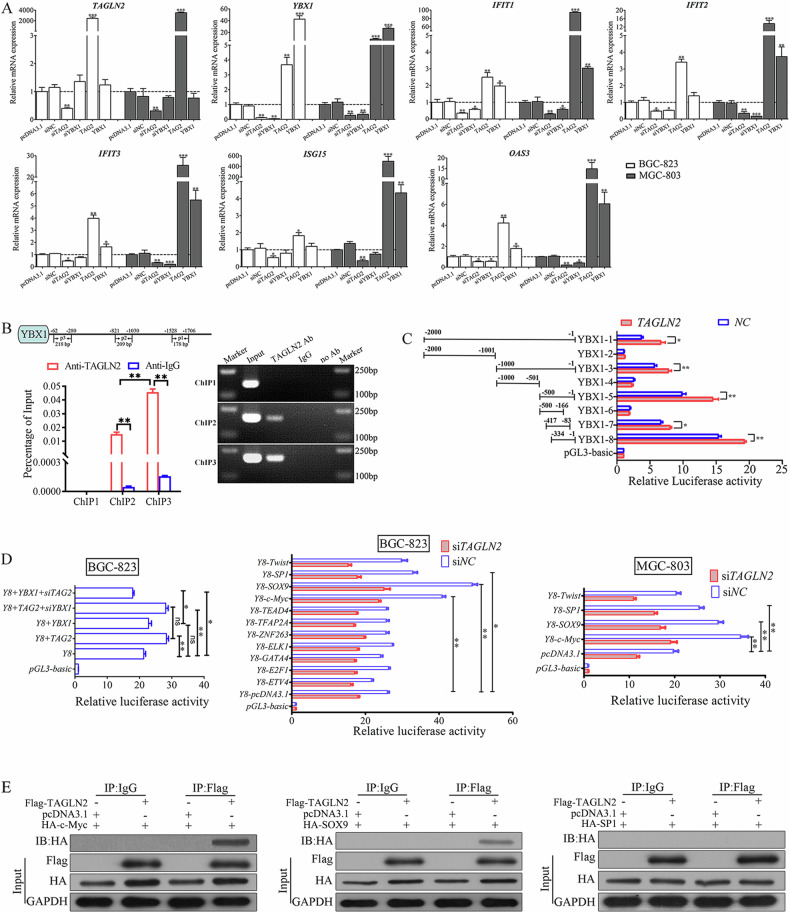
YBX1 plays a pivotal role in TAGLN2-induced ISG upregulation and is transcriptionally regulated by TAGLN2. **A** Quantitative RT‒PCR analysis confirmed the effect of YBX1 on TAGLN2-mediated ISG upregulation. **B** The endogenous interaction between TAGLN2 and the YBX1 promoter was evaluated by ChIP‒qPCR in BGC-823 cells. **C** The dual-luciferase reporter assay results showed that overexpression of TAGLN2 activated the transcriptional activity of YBX1 by binding to the promoter region (−334 to −1 bp). **D** The transcriptional regulation effect of YBX1 and 11 candidate transcription factors (ETV4, E2F1, GATA4, ELK1, ZNF263, TFAP2A, TEAD4, c-Myc, SOX9, SP1 and Twist) was evaluated by luciferase reporter assays in both BGC-823 and MGC-803 cells. *YBX1-8* promoter region was abbreviated as *Y8* for short. **E** Co-IP assays demonstrated that SOX9 and c-Myc, but not SP1, interacted with TAGLN2. *P < 0.05, **P < 0.01, ***P < 0.001.

RT‒qPCR analysis corroborated the link between YBX1 upregulation and ISGs elevation. *YBX1* expression levels rose or fell with the up- or downregulation of *TAGLN2*, respectively, in both BGC-823 and MGC-803 cells, with no inverse relationship observed (Fig. 3A). Furthermore, upregulation of five representative ISGs (*IFIT1, IFIT2, IFIT3, ISG15*, and *OAS3*) was observed in both *TAGLN2* and *YBX1* overexpression groups, with the increase in the *TAGLN2* overexpression group being notably more pronounced. Conversely, ISGs expression was significantly reduced in the *TAGLN2* or *YBX1* knockdown groups. These findings suggest YBX1 plays a pivotal role in TAGLN2-induced ISGs upregulation.

Chromatin immunoprecipitation (ChIP) followed by qPCR detected the endogenous interaction between TAGLN2 and the *YBX1* promoter. Agarose gel electrophoresis and sequencing results supported this finding (Fig. 3B). Cell cotransfection with a luciferase reporter driven by a fragment of the *YBX1* promoter region (YBX1-1~YBX1-8-Luc) and a TAGLN2 encoding expression vector, revealed that TAGLN2 overexpression significantly boosted YBX1’s transcriptional activity, particularly in the −334 to −1 bp promoter region (Fig. 3C). The YBX1-8 promoter was analyzed using the JASPAR database, and eleven candidate transcription factors were selected. YBX1-8-Luc was cotransfected with the *YBX1* expression vector or siRNA to rule out self-transcriptional regulation, given YBX1’s role as a critical transcription factor. The expression of the reporter was notably increased by three factors, namely SOX9, c-Myc, and SP1, in both BGC-823 and MGC-803 cells (Fig. 3D), with only SOX9 or c-Myc, but not SP1, interacting with TAGLN2 (Fig. 3E).

### YBX1 mediates TAGLN2-induced ISGs and PDL1 elevation via the cGAS-STING pathway

**Fig. 4:**
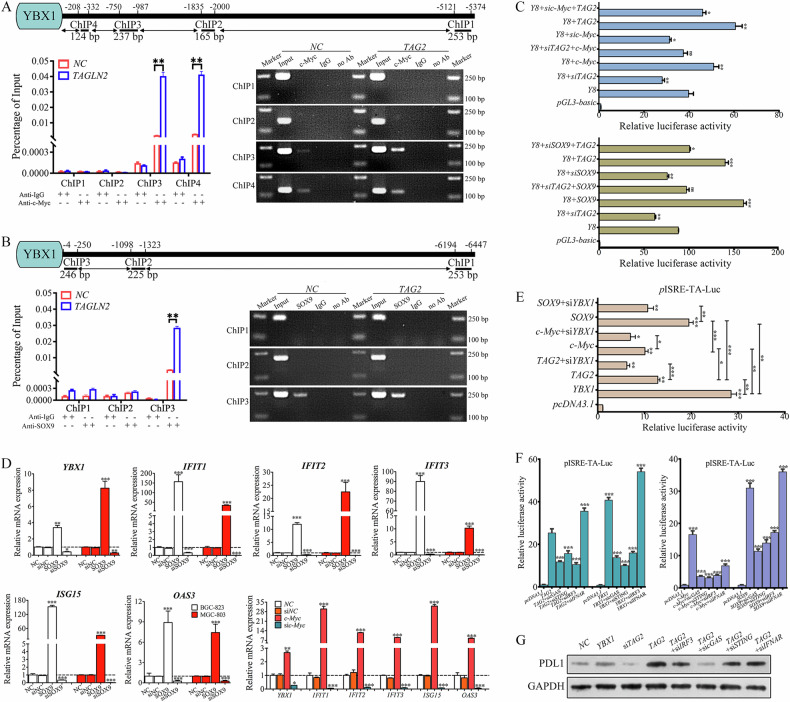
YBX1 mediates TAGLN2-induced ISGs and PDL1 elevation via the cGAS-STING pathway. **A**, **B** The endogenous interaction between SOX9 or c-Myc and the YBX1 promoter was identified by ChIP-qPCR and agarose gel electrophoresis. **C** The critical role of TAGLN2 in upregulating the expression of YBX1 at the transcriptional level was identified by luciferase reporter assay in MGC-803 cells. **D** Quantitative RT-PCR analysis showed that overexpression or downregulation of either c-Myc or SOX9 resulted in a substantial increase or decrease in the expression levels of YBX1 and five representative ISGs in both BGC-823 and MGC-803 cells, respectively. **E** Modulation of the expression of *c-Myc*, *TAGLN2*, *SOX9* or *YBX1* influenced the expression of pISRE-TA-Luc, as determined by luciferase reporter assay. **F** Luciferase reporter assay was used to analyze the expression of pISRE-TA-Luc by modulating the proteins involved in the cGAS-STING pathway, including *cGAS*, *STING*, *IRF3* and *IFNAR*. **G** Expression of PDL1 was detected in BGC-823 cells with *YBX1* or *TAGLN2* overexpression, or *TAGLN2* overexpression meanwhile si*cGAS*, si*STING*, si*IRF3* or si*IFNAR* treatment. *P < 0.05, **P < 0.01, ***P < 0.001.

The target regions for c-Myc (−987 to −1 bp and −332 to −1 bp, Fig. 4A) or SOX9 (−250 to −1 bp, Fig. 4B) were enriched approximately 17-fold or 49-fold over the negative control, respectively. The target region on the *YBX1* promoter for c-Myc or SOX9 overlapped with that for TAGLN2, and notably, the binding effect was further amplified by 27-fold for c-Myc or 34-fold for SOX9 with *TAGLN2* overexpression. As anticipated, YBX1-8-Luc expression was significantly increased after cotransfection with *TAGLN2, c-Myc*, or *SOX9* expression vectors (Fig. 4C). Conversely, expression significantly declined after cotransfection with *siTAGLN2, sic-Myc*, or *siSOX9*. Additionally, coexpression of *SOX9* or *c-Myc* with *siTAGLN2* treatment did not alter reporter expression, indicating that TAGLN2 mediates c-Myc or SOX9 binding to the *YBX1* promoter region. Coexpression of *TAGLN2* with *sic-Myc* or *siSOX9* also upregulated reporter expression, further confirming the transcriptional regulatory effects of c-Myc and SOX9. Collectively, these results underscore TAGLN2’s critical role in upregulating YBX1 expression at the transcriptional level by enabling c-Myc and SOX9 to bind to YBX1 promoter region. Overexpression of either *c-Myc* or *SOX9* substantially boosted the expression levels of *YBX1* and five representative ISGs in both BGC-823 and MGC-803 cells. Conversely, depleting either *c-Myc* or *SOX9* significantly inhibited the expression of these genes (Fig. 4D).

Compared to the control, overexpression of c-Myc, TAGLN2, SOX9, or YBX1 induced notably higher expression of pISRE-TA-Luc (IFN-stimulated response element), with the enhancement effect being strongest in the YBX1 overexpression group. Additionally, overexpression of c-Myc, TAGLN2, or SOX9 with YBX1 knockdown resulted in a significant decrease in ISRE activity. Notably, the YBX1-mediated regulation of ISRE-Luc expression was significantly more enhanced by SOX9 than c-Myc (Fig. 4E). The cytosolic DNA-sensing pathway was disrupted using siRNA targeted to *cGAS* (a key DNA sensor), *STING* (a messenger produced by cGAS in response to cytosolic DNA), or *IRF3* (transcription factors driving the transcription of genes encoding type I IFN and ISGs by binding to ISRE). The *TAGLN2*-induced expression enhancement of ISRE-Luc was prominently inhibited by si*cGAS*, si*STING*, or si*IRF3* treatment. Significant inhibitory effects were also observed in either YBX1-, c-Myc-, or SOX9-induced expression of ISRE-Luc with cytosolic DNA-sensing pathway disruption (Fig. 4F). However, si*IFNAR* (Type I IFN receptor) treatment further enhanced the expression promotion of ISRE by *TAGLN2*, *YBX1*, or *SOX9* overexpression, clarifying that TAGLN2-induced IFN signaling via cGAS-STING, not by creating more IFN production. Furthermore, inhibiting the extracellular IFN signaling transduction may block potential negative feedback mechanisms. Upregulation of PDL1 (Programmed Death Ligand 1) was induced by overexpression of YBX1 or TAGLN2, which was inhibited by si*cGAS*, si*STING*, or si*IRF3* but not by si*IFNAR* treatment (Fig. 4G). Therefore, YBX1-mediated TAGLN2-induced ISGs and PDL1 upregulation is driven by cytosolic ssDNA accumulation associated cGAS-STING pathway activation.

### TAGLN2 physically interacts with YBX1 and enhances its phosphorylation and nuclear translocation

**Fig. 5:**
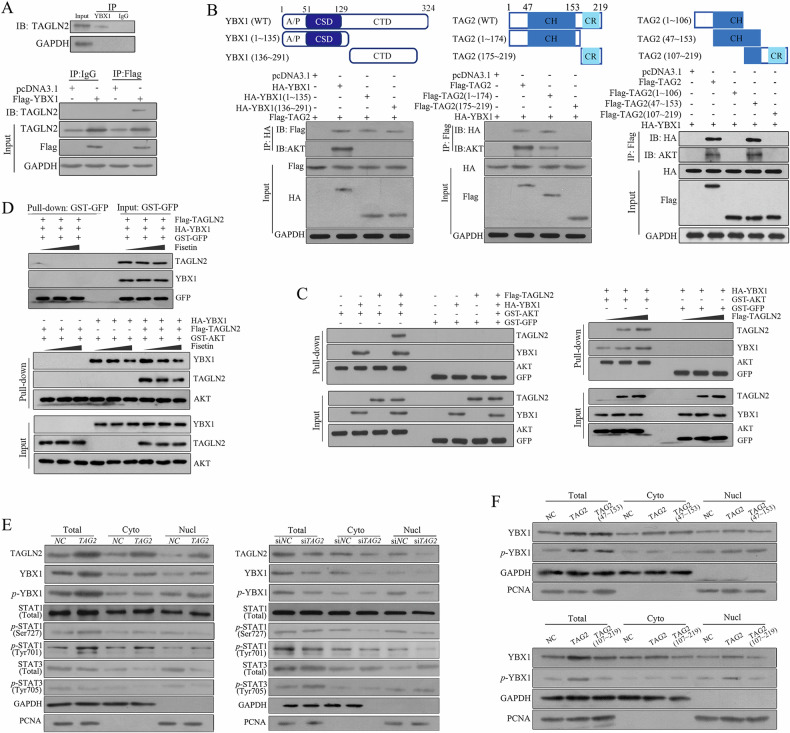
TAGLN2 physically interacts with YBX1 and enhances its phosphorylation and nuclear translocation. **A** The interaction between TAGLN2 and YBX1 was tested in both endogenous and exogenous Co-IP assays. **B** Various fragments of TAGLN2 and YBX1 were constructed to identify the molecular mechanism underlying the interaction of TAGLN2, YBX1 and AKT by Co-IP assay. **C** Purified HA-YBX1, Flag-TAGLN2 and GST-AKT proteins were prepared for the in vitro pull-down assay. An enhanced interaction between YBX1 and AKT was observed when the TAGLN2 protein input was increased from 100 μg to 200 μg. **D** Fisetin (2 nM or 4 nM) was used to suppress the interaction between AKT and YBX1. **E** TAGLN2 regulated the level of YBX1 phosphorylation and enhanced its cytoplasmic to nuclear translocation, followed by type I IFN activation. **F** Western blot analysis of total YBX1 and *p*-YBX1 for cytoplasmic and nuclear protein separation from BGC-823 cells transfected either with long-length TAGLN2, the fragment containing the complete CH domain (aa 47~153) or part of the CH domain (aa 107~219).

Endogenous TAGLN2 was observed in both the anti-YBX1 and anti-Flag (exogenous Flag-YBX1 overexpression) pull-down complexes, illustrating the interaction between TAGLN2 and YBX1 (Fig. 5A). Both the N-terminus containing the CSD domain and the C-terminus containing the CTD domain of *YBX1* had almost equal ability to interact with TAGLN2. Furthermore, only the fragments containing the complete CH domain (aa 47~153) of TAGLN2 were demonstrated to be responsible for the interaction with YBX1 (Fig. 5B). YBX1 has been shown to be a downstream substrate for AKT. As expected, AKT was detected in the HA-YBX1, Flag-TAGLN2, Flag-TAGLN2 (aa 1153) IP complexes, which all contained full-length YBX1 (Fig. 5B). These results suggested that YBX1 may mediate the interaction between TAGLN2 and AKT. Consistent with the Co-IP results, HA-YBX1 was pulled down with GST-AKT but not by GST-GFP, confirming a direct interaction between YBX1 and AKT by in vitro pull-down assay. However, Flag-TAGLN2 was only precipitated by GST-AKT in the presence of YBX1, indicating a YBX1-dependent interaction between TAGLN2 and AKT. Subsequently, a pull-down assay was conducted to assess whether the physical interaction between TAGLN2 and YBX1 could facilitate the association between AKT and YBX1. The findings demonstrated that the quantity of YBX1 precipitated by GST-AKT significantly increased from 1.6- to 2.7-fold when the TAGLN2 protein input was elevated from 100 to 200 μg (Fig. 5C). Fisetin was employed to hinder the interaction between AKT and YBX1. In the GST-AKT and HA-YBX1 interaction system, the binding affinity of YBX1 remained nearly constant upon the addition of 2 nM Fisetin but significantly diminished to 57% of its initial level with 4 nM Fisetin. In the AKT-YBX1-TAGLN2 interaction system, the binding affinity of YBX1 was reduced to 53% and 32%, and the binding of TAGLN2 was also reduced to 67% and 49% with 2 nM or 4 nM Fisetin, respectively. These outcomes imply that Fisetin attaches to the CSD of YBX1 and interrupts the interaction between TAGLN2 and YBX1, which in turn augments the inhibitory impact of Fisetin on the YBX1-AKT interaction (Fig. 5D).

It has been previously reported that YBX1, when phosphorylated by AKT, undergoes nuclear translocation from the cytoplasm. Our observations suggest that TAGLN2 amplifies the association between AKT and YBX1, leading us to investigate whether TAGLN2 influences the phosphorylation level of YBX1 and facilitates its nuclear translocation. Both total and phosphorylated YBX1 (*p*-YBX1) levels were markedly increased in *TAGLN2*-overexpressing cells, whereas they were notably decreased in si*TAGLN2* cells (Fig. 5E). After segregating cytoplasmic and nuclear proteins, it was observed that both cytoplasmic and nuclear levels of total YBX1 increased. However, only the nuclear, not the cytoplasmic, increase in *p*-YBX1 levels was evident when comparing the *TAGLN2* overexpression group to the control group. Consistently, nuclear, but not cytoplasmic, *p*-YBX1 levels were significantly reduced following si*TAGLN2* treatment, indicating that TAGLN2 modulates YBX1 phosphorylation and its subsequent recruitment into the nuclear compartment. Phosphorylation of STAT1 (*p*-STAT1) at Tyr701 is a well-established and sensitive indicator of type I IFN signaling activation. Overexpression of TAGLN2 led to a significant increase in STAT1 phosphorylation at Tyr701, but not at Ser727, and elevated both cytoplasmic and nuclear levels of *p*-STAT1 (Tyr701), while the total STAT1 levels remained unchanged. Similar results were observed following si*TAGLN2* treatment. An inverse trend was noted in the levels of STAT3 and *p*-STAT3 (Tyr705), aligning with prior findings that phosphorylated and unphosphorylated STAT3 competes with STAT1 for binding to the nuclear import protein, thereby suppressing downstream ISGs expression [20]. Furthermore, transfection with either full-length TAGLN2 or the fragment containing the complete CH domain (aa 47~153) was responsible for the increase in total YBX1 and *p*-YBX1 levels and subsequent nuclear translocation, and the fragment with part of the CH domain (aa 107~219) did not increase YBX1 and *p*-YBX1 levels (Fig. 5F).

### Disruption of the TAGLN2-YBX1-AKT interaction

**Fig. 6:**
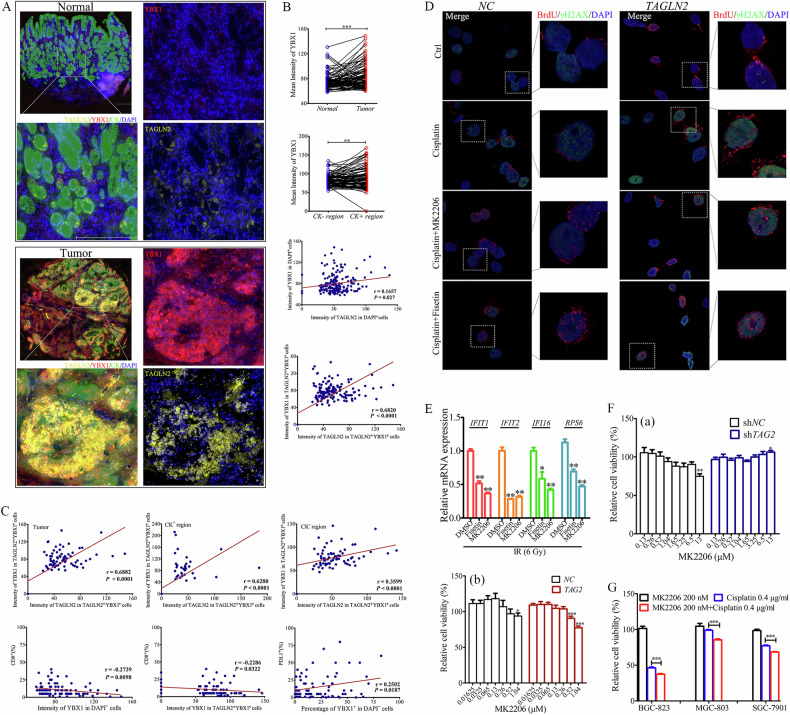
Disruption of the TAGLN2-YBX1-AKT interaction reduces cytosolic ssDNA accumulation, ISGs expression, and drug resistance. **A**~**C** Multiplex immunofluorescence of TMA was performed using the Opal 7-color Manual IHC Kit and VECTASHIELD^®^ HardSet Antifade Mounting Medium. The multiplex antibody panel was optimized as follows: TAGLN2, Opal 520 (yellow); CK, Opal 570 (green); YBX1, Opal 620 (red). The TMA was counterstained with DAPI (blue) and incubated with an antifluorescence quencher. Expression and spatial distribution of TAGLN2 or YBX1 in tissues and the correlation with patient clinical data. The DAPI channel was used to identify individual cells. A tissue segmentation algorithm combined with CK staining was applied to define tumoral and stromal areas. The scale bar is 200 μm. **D** Fisetin or MK2206 inhibited the accumulation of cytosolic ssDNA induced by overexpression of TAGLN2 by BrdU-γH2AX double labeling. HGC-27 cells stably transfected with TAGLN2 were prelabeled with BrdU and subsequently treated with 1 μg/ml Cisplatin with or without 10 μM Fisetin or 200 nM MK2206 in the medium. Cells were stained for DNA (DAPI, blue), the primary BrdU antibody (red) and phospho-histone H2AX (green). **E** Relative mRNA levels of the panel of IFN-related genes with or without 10 μM Fisetin or 200 nM MK2206 in the medium after 6 Gy X-ray treatment were evaluated by quantitative RT‒PCR analysis. The cytotoxicity induced by MK2206 from 16.25 nM to 13 μM (**F**) or MK2206 (200 nM) and Cisplatin (0.4 μg/ml) combination on tumor cells was detected (**G**). *P < 0.05, **P < 0.01, ***P < 0.001.

To investigate the spatial distribution of TAGLN2 and YBX1 in GC tissue, a multiplex immunofluorescence panel for TAGLN2, YBX1, CK, and DAPI was utilized to analyze 90 tumor tissues and their paired normal counterparts. The mean intensity of TAGLN2 and YBX1 in DAPI^+^ cells was significantly higher in tumor tissues than in paracancerous tissues (Fig. 6A, B). More TAGLN2 accumulation was observed in stromal areas (CK^-^ region) than in tumoral areas (CK^+^ region) of tumor tissues. However, YBX1 accumulated more in tumoral areas than in stromal areas. Moreover, there was a close positive correlation between the expression of TAGLN2 and YBX1 in DAPI^+^ cells of both tumor and paracancerous tissues (*r* = 0.1657, *P* = 0.027). Furthermore, the correlation became more significant when focusing on the cells with TAGLN2- and YBX1-positive signals (TAGLN2^+^YBX1^+^) in all tumor and paired control tissues (*r* = 0.6820, *P* < 0.0001) and only tumor tissues (*r* = 0.6882, *P* < 0.0001). The correlation was higher in the CK^+^ region (*r* = 0.6280, *P* < 0.0001) than in the CK^-^ region of tumor tissues (*r* = 0.3599, *P* < 0.0001). The expression of YBX1 in DAPI^+^ cells (*r* = −0.2739, *P* = 0.0098) or in TAGLN2^+^YBX1^+^ cells (*r* = −0.2286, *P* = 0.0322) was negatively correlated with the percentage of CD8^+^ cells in the tumor region but positively correlated with the percentage of PDL1^+^ cells (*r* = 0.2502, *P* = 0.0187, Fig. 6C).

Significant accumulation of cytosolic BrdU foci, which was markedly induced by Cisplatin, was substantially reduced by either Fisetin (10 μM) or MK2206 (200 nM) treatment (Fig. 6D) in *TAGLN2*-overexpressing cells. The expression levels of ISGs were significantly inhibited by Fisetin or MK2206 under 6 Gy irradiation treatment (Fig. 6E). After culturing *TAGLN2*-knockdown or overexpressing cells with a series of MK2206 concentrations ranging from 16.25 nM to 13 μM, the results demonstrated that the higher the intracellular TAGLN2 protein level, the stronger the cytotoxicity induced by MK2206 (Fig. 6F). The combination of MK2206 (200 nM, a low concentration with almost no cytotoxicity) and Cisplatin (0.4 μg/ml) significantly enhanced the cytotoxicity of low-concentration Cisplatin on tumor cells (Fig. 6G). These results confirmed that disruption of the TAGLN2-YBX1-AKT interaction reduces cytosolic ssDNA accumulation, ISGs expression, and drug resistance.

### Potential clinical value of TAGLN2-associated AKT-YBX1-ISGs axis

**Fig. 7:**
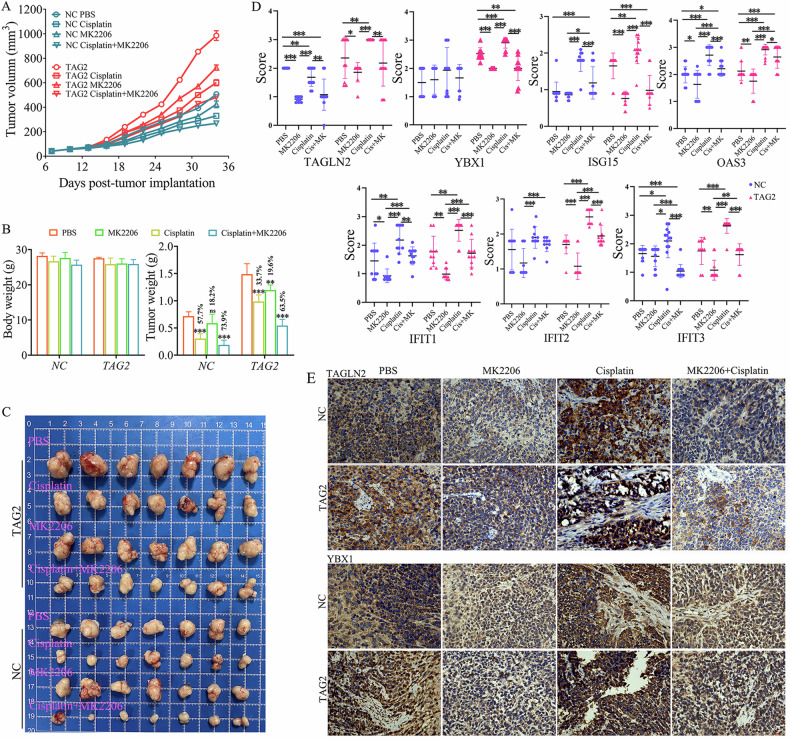
Inhibiting AKT-YBX1-ISGs axis enhances the sensitivity of gastric cancer xenografts to the Cisplatin treatment. **A**–**C** A subcutaneous mouse model was established to confirm the effects of TAGLN2 and associated axis on tumor growth in vivo. The mice were injected subcutaneously with 2 × 10^6^ BGC-823 cells with (NC group) or without TAGLN2 overexpression (TAGLN2 group) in 0.2 ml of PBS. Intraperitoneal administration of Cisplatin (3 mg/kg, every 4 days), MK2206 by intragastric gavage (120 mg/kg, every 4 days), or a combination of both (MK2206 was administrated 2 days before Cisplatin administration), or PBS was given in control group. Tumor volume, body weight and tumor weight were measured. (D&E) Representative protein set (TAGLN2, YBX1, IFIT1, IFIT2, IFIT3, OAS3 and ISG15) staining in tumors derived from NC or TAGLN2 group with Cisplatin, MK2206, or a combination of both treatment. The results were obtained by multiplying the PP by the IS (score = PP × IS). *P < 0.05, **P < 0.01, ***P < 0.001. The scale bar is 10 μm.

A subcutaneous mouse model was established to confirm the effects of TAGLN2 and associated axis on tumor growth in vivo (Fig. 7A–C). In contrast to NC group, TAGLN2-overexpressing group exhibited faster tumor growth and larger tumors. Cisplatin or MK2206 treatment showed retarded tumor growth; moreover, combination therapy of the two showed a stronger inhibitory effect both in NC and TAGLN2-overexpressing group. Body weight was not affected by the treatment. Through the results of tumor weight, it was found that TAGLN2-overexpressing group exhibited higher tolerance to Cisplatin treatment than NC group (33.7% vs 57.7%), and the inhibitory ability of MK2206 treatment alone was similar (19.6% vs 18.2%). However, the combination of the two drugs produced a synergistic effect in the TAGLN2-overexpressing group (inhibition rate increased about 10%), and MK2206 enhanced the sensitivity of higher *TAGLN2*-expressing tumors to Cisplatin treatment (Fig. 7B). Histological analyses of mouse tumors revealed that the expression of a protein set (TAGLN2, YBX1, IFIT1, IFIT2, IFIT3, OAS3, and ISG15) in the TAGLN2-overexpressing group was higher than that in NC group and that the expression of the protein set was further elevated after Cisplatin treatment. Furthermore, the increase in expression of the protein set was significantly eliminated by additional MK2206 use (Fig. 7D, E) in the TAGLN2-overexpressing group. But the effect of MK2206 on the control group was relatively moderate. Overall, these findings suggest that inhibiting the AKT-YBX1-ISGs axis enhances the therapy sensitivity of tumors with higher TAGLN2 levels.

**Fig. 8:**
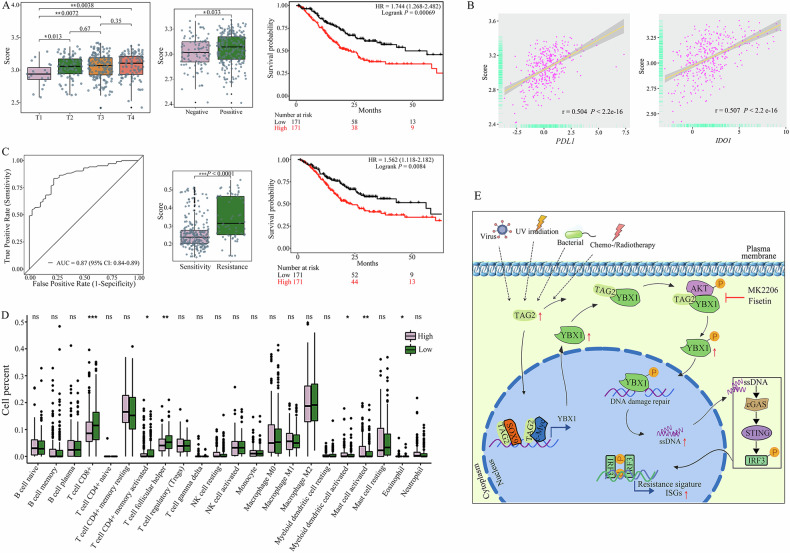
Potential clinical value of TAGLN2, YBX1, and TAGLN2-associated ISGs in GC. **A** Clinical analysis of the experimentally derived nine-gene pair (*IFIT1, IFIT2, IFIT3, ISG15, IFI16, OAS3, WARS, YBX1, TAGLN2*). The survival curves were plotted by the Kaplan‒Meier method and tested by log-rank analysis. The clinical characteristics of patients with different protein expression levels were compared by using a two-tailed chi-square test with SPSS 20 software. **B** Expression of IRDS genes also positively correlated with the expression of *PDL1* or *IDO1*, respectively. **C** The RandomForest algorithm based on Python sklearn was used to construct the model, and receiver operating characteristic (ROC) curve analysis was used to evaluate the predictive accuracy and sensitivity of the therapy prediction model. Outcomes were divided into therapy sensitivity (including CR complete remission, PR partial remission/response, SD stable disease) and therapy resistance (PD progressive disease) based on the data of “primary_therapy_outcome_success”. **D** Immune cell proportion analysis. Formatted data were uploaded to the CIBERSORT web portal to analyze immune cell proportions (https://cibersort.standord.edu./). *P < 0.05, **P < 0.01, ***P < 0.001. **E** Schematic diagram of the role of TAGLN2-mediated AKT-YBX1 pathway activation in IFN-related DNA damage resistance.

Since the identified IRDS gene set (*IFIT1, IFIT2, IFIT3, ISG15, IFI16, OAS3, WARS, YBX1, TAGLN2*) was experimentally proven to be highly correlated and functionally related in this study, its potential clinical value was further evaluated. The results showed that the expression of IRDS genes was significantly positively correlated with T stage and positive lymph nodes, and high expression of IRDS genes predicted poor overall survival outcomes (Fig. 8A). Expression of IRDS genes also positively correlated with the expression of *PDL1* (*r* = 0.504) or *IDO1* (*r* = 0.507), respectively (Fig. 8B). The AUC value of the model for predicting the primary therapy outcome of patients who received chemo- or/and radiotherapy was 0.87 (95% CI: 0.84–0.89). Based on the model, the patients in the therapy sensitivity group had lower IRDS expression and better overall survival outcomes than those in the therapy resistance group (*P* < 0.0001) (Fig. 8C). Moreover, high IRDS gene expression was associated with an immunosuppressive phenotype characterized by a low abundance of CD8^+^ T cells, activated memory CD4^+^ T cells, and T follicular helper cells (Fig. 8D). Overall, these results suggest that this nine-gene model is effective for predicting therapeutic resistance and long-term prognosis in GC patients with different IRDS statuses.

## Discussion

Here, we show that high expression levels of TAGLN2 in GC are associated with IFN-related DNA damage resistance. In previous studies, TAGLN2 was mainly recognized as an actin stress fiber-associated protein involved in actin filament stabilization. However, recent reports have demonstrated that TAGLN2 is associated with cancer progression, migration, invasion and prognosis. We previously clarified that TAGLN2 also plays an important role in promoting tumor angiogenesis by activating NRP1/VEGFR2 and downstream MAPK signaling pathways [21]. Moreover, accumulating evidence has clarified that elevated TAGLN2 levels contribute to drug resistance. TAGLN2 overexpression activated the PI3K/Akt/GST-3β pathway and downregulated the expression of PTEN, which promoted paclitaxel treatment resistance and the migration and invasion of breast cancer cells [18]. However, the precise biological role and underlying mechanism of TAGLN2 in tumor therapy resistance is unclear.

Our work has presented a number of novel features that suggest the importance of TAGLN2 in GC therapy resistance. Our results indicate that TAGLN2-induced ISGs upregulation contributes to the generation of ssDNA that accumulates in the cytosol following TAGLN2 overexpression. We observed aberrantly upregulated TAGLN2 expression in the early stage of GC and TAGLN2 expression levels continuously increased with tumor progression. Consistent TAGLN2 expression triggered the upregulation of downstream resistance signature ISGs by activating AKT-YBX1 signaling with dual pathways, followed by cytoplasmic ssDNA aggregation-mediated IFN signal activation (Fig. 8E).

However, the causes of TAGLN2 expression upregulation are still unclear. In previous reports, the expression of TAGLN2 has been identified to be significantly upregulated upon stimulation with IgM, bacterial endotoxin LPS or hepatitis B-encoded X protein (HBx), suggesting that its expression is triggered by external inflammatory signals. Approximately 12% of human cancers can be caused by pathogens, including *Helicobacter pylori*, human papillomavirus (HPV), hepatitis B virus (HBV), hepatitis C virus (HCV), Epstein-Barr virus (EBV), human immunodeficiency virus (HIV) and human herpesvirus 6 (HHV) [9]. *Helicobacter pylori* accounts for more than 60% of GC cases. In addition to pathogen infection, we also found that the mRNA expression of *TAGLN2* was upregulated by irradiation treatment at both 4 Gy or 6 Gy. DNA-damaging agents, including UV irradiation and ionizing radiation, can cause cancer. Therefore, consistent exposure to a low level of infection or external radiation can induce lasting expression of TAGLN2. Our study showed that aberrantly upregulated TAGLN2 expression triggered the elevation of downstream resistant signature ISGs through cytoplasmic ssDNA aggregation-induced IFN signal activation. In contrast to “emergency” infection or exposure, “physiological” continuous low levels of IFNs were able to induce steady-state upregulation of resistance signature ISGs in cancer cells, with no increase in proapoptotic and antiproliferative ISGs predominantly expressed by intratumoral immune cells [9, 12]. Only approximately 30 ISGs out of more than 100 are known to be consistently overexpressed in therapy-resistant tumors, including *IFI27, ISG15, BST2 OAS1, OAS3, and OASL*. Most of them were identified in breast cancer cells that were resistant to chemo- and radiation therapy, but there are still no data on the ISG overexpression in GC cells. In our study, ISGs, including *IFIT1, IFIT2, IFIT3, ISG15, OAS3, IFI16, WARS* and *TAP1*, were identified in GC cells for the first time. Therefore, chronic exposure to infection or external radiation led to persistent TAGLN2 expression, which induced cytoplasmic ssDNA aggregation and triggered continuous low levels of IFN-activated resistance signature ISG upregulation in GC patients. Cancer cells acquire inflammatory memory is associated with prolonged IFN signaling and a subset of ISGs [8]. Moreover, persistent TAGLN2 expression might simulate a “pathological status of chronic infection”, and mediates immune-suppressive phenotype, which is likely to provide a new perspective for how cancer cells retain effects of prolonged IFN stimulation.

Our study contributes to accumulating literature linking YBX1-associated damage repair processing to IFN-related DNA damage resistance. As a nuclease-sensitive element-binding protein 1, YBX1 (Y-box-binding protein) is a multifunctional oncoprotein that plays important roles in regulating transcription, translation, mRNA splicing and DNA repair [22], and its expression is regulated by E-box binding transcription factors such as c-Myc, Twist and Math2. YBX1 is involved in many important processes of tumor development, including proliferation, invasion, metastasis, tumor stem cell characteristics, chemo- and radiotherapy resistance, and DNA damage repair. However, the use of YBX1 as a therapeutic target or biomarker is still of limited concern. YBX1 has predictive value for drug resistance and poor prognosis in more than 20 cancer types, and upregulates the expression of multiple drug resistance genes, including *ABCB1, MVP/LRP, TOP2A, CD44, CD49f, BCL2,* and *MYC* [23], upon UV irradiation or Cisplatin treatment. YBX1-associated PDL1 upregulation aids tumor immune escape [24]. YBX1 promotes DNA damage repair in through multiple processes, such as preferentially binding to Cisplatin-modified DNA, promoting the separation of double-stranded DNA substrates containing base mismatches, preferentially binding to single-stranded nucleic acids, exhibiting 3′-5′ exonuclease activity, exhibiting endonuclease activity on double-stranded DNA, and interacting with PCNA, MSH2, Ku80, WRN and DNA polymerase δ proteins to participate in multiple DNA damage repair processes [25]. LncRNA HCP5 recruited YBX1 to bind to the promoter of the mismatch repair gene MSH5 to regulate its transcription and DNA damage repair [26]. Specifically, the lncRNA HCP5 inhibited ribosomal S6 kinase (RSK) and downstream AKT expression, reduced YBX1 phosphorylation, blocked the repair of double-stranded DNA breaks after radiation, and transcriptionally inhibited the expression of the drug resistance target genes MDR1 and MVP [27, 28]. In summary, the important function of YBX1 in DNA repair and in conferring drug resistance in tumor cells is the key theoretical support of our study. In our study, we report a new function of YBX1 as an upstream mediator of the cytosolic DNA-sensing pathway and TAGLN2-induced IFN-related DNA damage resistance for the first time.

Our work is consistent with previous reports on breast cancer, linking high expression of IRDS genes to clinical chemoradio resistance, which might be used as therapy-predictive markers. In our study, nine IRDS genes (*IFIT1, IFIT2, IFIT3, ISG15, IFI16, OAS3, WARS, YBX1, TAGLN2*) were selected based on an experimentally derived gene expression profile. In contrast to the results from currently popular computational methods for estimating hub genes, there are close regulatory relationships among these predictive markers. Our work provides potential therapeutic intervention or sensitization strategies for cancer patients with upregulated IRDS gene expression. Expression of IRDS genes in cells was significantly downregulated with the following treatments: inhibited YBX1 phosphorylation (treatment with Fisetin to disrupt AKT-YBX1 interaction, or with MK2206 to selectively inhibit the phosphorylation of AKT), downregulated the expression of TAGLN2 or YBX1 (caused by si*TAGLN2*, si*YBX1*, si*SOX9*, or si*c-My*c, respectively), or disrupted the downstream cGAS-STING pathway (caused by si*cGAS*, si*STING* or si*IRF*, respectively*)*. It is noteworthy that SOX9 is one of the critical transcription factors involved in many diseases, including cancer, and increasing evidence shows that SOX9 is an emerging driver of cancer drug resistance [29]. Fisetin has been proven to regulate the PI3K/AKT/mTOR, Wnt/β-catenin, NF-κB and TRAIL/TRAIL-R pathways to inhibit metastatic spread, autophagy, necroptosis and angiogenesis in different cancer types. It has remarkable potential as a chemopreventive agent and is currently being tested in various phases of clinical trials [30]. In our study, Fisetin not only suppressed the interaction between AKT and YBX1 but also bound to the CSD domain of YBX1 to disrupt the interaction of TAGLN2 and YBX1, which subsequently enhanced the inhibitory effect of Fisetin on the YBX1-AKT interaction. Therefore, Cisplatin-induced accumulation of cytoplasmic ssDNA in *TAGLN2*-overexpressing cells was markedly reduced by Fisetin treatment, suggesting potential sensitization strategies for IFN-related DNA damage-resistant tumors.

Moreover, PDL1 is largely induced by IFNγ, which is mainly produced by differentiated effector T cells in the tumor tissue [31]. We observed that upregulation of PDL1 was induced by overexpression of YBX1 or TAGLN2, and YBX1 were positively correlated with the percentage of PDL1^+^ in GC; therefore, elevated expression of the TAGLN2-YBX1-ISGs axis proteins might resensitize IFN-related DNA damage-resistant tumors to PD-1/PDL1-targeting immune checkpoint blockade (ICB) therapy.

In conclusion, our study contributes to the accumulating literature linking TAGLN2-mediated AKT-YBX1 pathway activation to IFN-related DNA damage resistance by recruiting the transcription factors c-Myc and SOX9 to the promoter of YBX1 and directly interacting with YBX1 to enhance its phosphorylation and nuclear translocation in dual ways. Our work suggests that TAGLN2, YBX1 and induced ISGs are novel predictive markers for clinical outcomes, and that targeting TAGLN2-mediated ISG upregulation is an attractive therapeutic sensitization strategy for gastric cancer patients.

## Materials and methods

### Cells and culture

AGS, KATOIII, NCI-N87, SNU-1, SNU-5, SNU-16, and HS-746T cell lines were purchased from ATCC (Manassas, USA). HGC-27, BGC-823, MGC-803, MKN-45, MKN-28, SGC-7901 and GES-1 cell lines were purchased from the Institute of Cell Biology (Shanghai, China). Cells were cultured in RPMI 1640 or DMEM supplemented with 10% fetal bovine serum (FBS, Sigma, USA), 100 U/ml penicillin and 100 μg/ml streptomycin according to recommendations. Plasmids or siRNAs were transfected into cells with Lipofectamine 2000 or Lipofectamine™ RNAi MAX (Thermo, USA), respectively. Stably transfected clones were infected with lentiviral expression vector and established by G418 selection.

### Tissue microarrays (TMA) and patient cohorts

TMA of GC and paired normal tissues were purchased from Shanghai Outdo Biotech Company (Shanghai, China). Cohort 1 (HstmA150CS02) contained 75 tumor tissues, and the tumor tissues and their paired normal counterparts were used for immunohistochemical staining (IHC) [21]. Cohort 2 (HstmA180Su11) contained 90 tumor tissues, and the tumor tissues and their paired normal counterparts were used for multiplex immunofluorescence. The detailed clinicopathological characterization of cohort 2 is summarized in Supplementary Table 4.

### ITRAQ-2DLC‒MS/MS and RNA-seq analysis

Full-length cDNA encoding human *TAGLN2* was amplified by PCR and cloned into the pcDNA3.1 vector. Small interfering RNA targeting *TAGLN2* was synthesized by Sigma. Primers for plasmid construction or siRNA target sequences were listed in Supplementary Tables 5 and 6. Effective gene knockdown was achieved at 50 nM of si*TAGLN*2#3. BGC-823 cells transiently transfected with pcDNA3.1-*TAGLN2*, si*TAGLN2*#3 or mock control (three replicates per group) were harvested after a 48-hour transfection, respectively. Cells were lysed, and proteins were digested and labeled with iTRAQ reagents following the manufacturer’s instructions (AB Sciex, USA). The parameters for iTRAQ-2DLC‒MS/MS analysis, protein searches, and quantification were set according to the method described in our previous report [32]. Total RNA was extracted, mRNA libraries were sequenced on an Illumina sequencing platform, and bioinformatic analysis was performed by Genedenovo Biotechnology Co., Ltd (Guangzhou, China).

### In vitro assays of the therapeutic resistance function of TAGLN2

Cells with altered TAGLN2 expression were grown in 96-well plates (100 μl, 2 × 10^3^/well) and treated with Cisplatin at concentrations of 0.2, 0.4, 0.8, 1.6 and 3.2 μg/ml for 24 h (BGC-823 cells) or 48 h (HGC-27 cells), Oxaliplatin at concentrations of 0.16, 0.32, 0.64, 1.28, 2.56, 5.12, 10.24 and 20.48 μM, 5-Fu at concentrations of 5, 10, 20, 40, 80, 160, 320, 640 and 1280 μM, Capecitabine at concentrations of 4.8, 2.4, 1.2, 0.6, 0.3, 0.15, 0.075, 0.0375, 0.01875 mM, or Doxorubicin (Dox) at concentrations of 1, 2, 4 μM for 48 h. Cell viability was determined using Cell Counting Kit-8 (CCK-8) according to the manufacturer’s instructions. The IC_50_ was calculated based on the absorbance report.

BGC-823 cells with or without *TAGLN2* knockdown were irradiated with 0, 4, and 6 Gy X-ray radiation (RadSource-2000 X-ray Irradiator). Cell viability and colony formation ability were measured by CCK-8 and colony formation assays. For the cell colony formation assay, cells were seeded on 6-well plates at a density of 1 × 10^3^ cells per well and cultured for 2 weeks. The colonies were fixed with methanol and stained with 0.5% crystal violet in 50% methanol. Colonies larger than 100 μm in diameter were counted.

### BrdU-γH2AX double labeling

Cells were cultured with 10 μg/ml BrdU (BD Bioscience, Cat#550891) in RPMI 1640 containing 10% FBS for 36 h. After washing with PBS, the cells were returned to BrdU-free medium. Then, the cells were treated with 1 μg/ml Cisplatin for 2, 4, or 8 h, with or without 10 μM Fisetin or 200 nM MK2206 added to the medium (cat #S2298 and S1078, Selleck, USA). For immunofluorescence staining, cells were permeabilized with CSK buffer (10 mM PIPES at pH 6.8, 300 mM sucrose, 100 mM NaCl, 1.5 mM MgCl_2_, 0.5% Triton) on ice for 2 min and then fixed with 4% paraformaldehyde at room temperature for 10 min [14]. Samples were blocked with 10% FBS in PBS for 1 h and incubated with the primary antibodies BrdU and phospho-histone H2AX (S139) (R&D Systems, Cats#MAB7225 and AF2288) for 3 h and with secondary antibodies for 1.5 h at room temperature. Three washes for 5 min each in PBS were used to remove unbound antibodies. Slides were mounted with VectaShield antifade mounting medium with DAPI (Vector Laboratories). Images were taken using a 63× objective on a Zeiss LSM780 confocal microscope equipped with the Axio Observer Z1 platform (Carl Zeiss GmbH, Oberkochen, Germany).

### RNA extraction and quantitative RT-PCR

Total RNA was extracted using TRIzol (Invitrogen, USA), and consecutive reverse transcription to cDNA was performed using the EasyScript First-Strand cDNA Synthesis SuperMix kit (TransGen, China) according to the manufacturer’s instructions. Reactions were performed using AceQ qPCR SYBR Green Master Mix (Vazyme, China) in an ABI PRISM® 7500 Sequence Detection System. All reactions were performed at least in triplicate. The sequences of the forward and reverse primers are provided in Supplementary Table 7.

### Chromatin Immunoprecipitation (ChIP)

ChIP was performed with one of the following antibodies: TAGLN2 (Affinity Biosciences, Cat# DF12053), c-Myc (Abcam, Cat# ab32072), or SOX9 (Abcam, Cat# ab185230). A nonspecific rabbit IgG antibody was used as a negative control in BGC-823 cells. A Pierce ChIP assay kit (Thermo Fisher Scientific) was used according to the manufacturer’s instructions. The precipitated DNA was subjected to RT‒qPCR and agarose gel electrophoresis. ChIP-qPCR primer pairs that overlap the human YBX1 promoter region were designed for TAGLN2, c-Myc, and SOX9 (Supplementary Table 8).

### Dual-luciferase assay

Unequal lengths of *YBX1* gene promoter region YBX1-1 (−2000 to −1 bp), YBX1-2 (−2000 to −1001 bp), YBX1-3 (−1000 to −1 bp), YBX1-4 (−1000 to −501 bp), YBX1-5 (−500 to −1 bp), YBX1-6 (−500 to −166 bp), YBX1-7 (−417 to −83 bp) and YBX1-8 (−334 to −1 bp) were used for the luciferase reporter assays and were amplified by PCR. Primer pair sequences are presented in Supplementary Table 9. The PCR fragments were then cloned into the *Mlu*l/*Xho*I sites of the pGL3-basic vector and verified by sequencing. The pGL3-YBX1~8 plasmids were transiently transfected into BGC-823 cells or MGC-803 cells with multiple differentially expressed genes. The activity of the empty vector pGL3-basic was used for comparison. Luciferase activity was measured following the Dual-Luciferase Reporter Assay protocol (Promega).

### Coimmunoprecipitation (Co-IP) assay

Co-IP was performed using a Pierce Co-IP kit (Cat#26149, Thermo Fisher Scientific). The manufacturer’s protocol was followed to bind anti-TAGLN2, anti-SOX9, anti-c-Myc, anti-SP1 (Abcam, Cat# ab124804), anti-YBX1 (ProteinTech, Cat# 20339-1-Ap), anti-Flag, or anti-HA antibodies to the antibody coupling resin. Protein lysates from cells were added to the respective antibody-bound column and incubated overnight with gentle shaking at 4 °C. The next day, the bound immune complex was recovered by centrifugation and washed 5 times with washing buffer. After placing the spin column into a new collection tube, elution and centrifugation steps were repeated more than twice as needed, and flow-through was collected. The lysates and immunoprecipitates were detected by mass spectrometry analysis or Western blot using relative antibodies. To identify the molecular mechanism of the interaction between TAGLN2 and YBX1, wild-type or TAGLN2 fragments (aa 1 to −174, 175 to −219, 1 to −106, 47 to −153, 107 to −219) and wild-type or YBX1 fragments (aa 1 to −135, 136 to −291) were constructed in the pcDNA3.1 vector. The association of Flag-tagged TAGLN2 with HA-tagged YBX1 was determined using Co-IP in transfected BGC-823 cells.

### GST pull-down assay

The recombinant proteins including GST-AKT, GST-GFP, HA-YBX1 and Flag-TAGLN2 were expressed in *E. coli* using expression vector pET-28a (+) and pGEX-4T-2. The Pierce^TM^ GST Protein Interaction Pull-Down Kit (Thermo Fisher Scientific, Cat#21516) was used according to the manufacturer’s instructions. GST-tagged fusion protein was immobilized on equilibrated glutathione agarose resin at 4 °C for 2 h with gentle rocking motion. After centrifugation and washing 5 times with washing buffer, the spin column containing the immobilized GST-tagged bait protein was incubated with prepared prey protein samples (100 μg HA-YBX1 mixed with 100 or 200 μg TAGLN2) at 4 °C for 2 h, and then the wash steps were repeated. For inhibited interaction observation, 2 or 4 nM Fisetin was added to the mixture. The resin was eluted with glutathione elution buffer, and the protein samples were evaluated via Western blot.

### Nuclear and cytoplasmic protein extraction

To evaluate the expression of TAGLN2, total and phospho-YBX1, STAT1, or STAT3 in nuclei, nuclear proteins were isolated from harvested cells by a nuclear and cytoplasmic protein extraction kit (Beyotime, China) following the manufacturer’s protocol. Western blot was carried out to detect proteins with the following antibodies: TAGLN2, YBX1, phospho-YBX1 (Ser102) (Affinity Biosciences, Cat#AF8525), STAT1 and phospho-STAT1 (Tyr701) (Abcam, Cats#ab109320 and ab30645), and STAT3 and phospho-STAT3 (Tyr705) (CST, Cats#12640 and 9145). A PCNA antibody (CST, Cat#13110) was used as a loading control for the nucleus, and a GAPDH antibody was used as an internal control for the cytoplasm.

### Establishment of xenograft model and treatment

BALB/c nude mice (male, 6-week-old) were maintained and treated under specific pathogen-free conditions, and were performed in accordance with the guidelines. The mice were injected subcutaneously with 2 × 10^6^ BGC-823 cells with or without TAGLN2 overexpression in 0.2 ml of PBS. Mice were randomly allocated to different experimental groups. Tumor volume and body weight were measured every 3 days for the duration of the experiment. Tumor volume was calculated using the following equation: tumor volume = d1 × (d2)^2^ × 0.5, where d1 is the largest diameter and d2 is the perpendicular diameter. The mice were monitored daily for survival.

For radiation treatment, when the volume reached 60–100 mm^3^, tumors were exposed to a single dose of 250 cGy radiation with a cycle of 4 days under two exposure, 6-day interval, and another two exposure. For drugs treatment, mice were sorted into 4 experimental groups with 7 mice per group as follows: intraperitoneal administration of Cisplatin (3 mg/kg, every 4 days), MK2206 by intragastric gavage (120 mg/kg, every 4 days), or a combination of both (MK2206 was administrated 2 days before Cisplatin administration), or PBS was given in control group. Mice were sacrificed, and tumors were excised, weighed and snap-frozen in liquid nitrogen for further histological analysis.

### Immunohistochemical staining

IHC staining was performed using EnVision + System-HRP kits. Analysis/scoring of IHC data was performed by two certified pathologists at 40× magnification from our hospital, and the scores were averaged. The percentage of positive (PP) cells was scored as 0 (PP ≤ 5%), 1 (6% ≤ PP ≤ 25%), 2 (26% ≤ PP ≤ 50%), 3 (51% ≤ PP ≤ 75%), and 4 (PP ≥ 75%). The intensity of staining (IS) was scored as 0: negative, 1: weak, 2: moderate, 3: strong and 4: very strong. The results were obtained by multiplying the PP by the IS (immunoreactive score = PP × IS).

### Multiplex immunofluorescence

Multiplex immunofluorescence of TMA was performed using an Opal 7-color Manual IHC Kit (NEL801001KT, PerkinElmer, Waltham, USA) and VECTASHIELD^®^ HardSet Antifade Mounting Medium (H-1400, Vector Labs) according to the following staining steps. After deparaffinization in xylene, rehydration through graded alcohols, and heat treatment for antigen retrieval, slides were then blocked and stained with one of the following antibodies to process immunofluorescence staining for 1 h: TAGLN2 (1:500), YBX1 (1:4000, Novus Biologicals), and CK (1:2, Abcarta Medtech Co., Ltd, China). Next, the TMA were incubated with HRP-labeled secondary antibody for 10 min. Then, Opal dye working buffer was added and incubated at room temperature for 10 min. The staining steps were repeated until every antigen was labeled by distinct Opal fluorophores. The immunofluorescence signal was amplified by the tyramide signal amplification (TSA) technique. Heat treatment was performed to remove the antibody-TSA complex for every staining cycle. The multiplex antibody panel was optimized as follows: TAGLN2, Opal 520; CK, Opal 570; YBX1, Opal 620. The TMA were counterstained with DAPI and incubated with an antifluorescence quencher.

### Image acquisition and digital image analysis

Panoramic multispectral scanning and quantitation of TMA were achieved on the TissueFAXS Spectra Systems and StrataQuest analysis software (TissueGnostics). The spectral library for spectral splitting was established to eliminate the interference of cross-fluorescence. The DAPI channel was used to identify individual cells, and then the expression of TAGLN2 or YBX1 was computed based on the positively stained cells. A tissue segmentation algorithm combined with CK staining was applied to define tumoral and stromal areas. The distance radius was set according to the staining of each protein channel, and the threshold was set according to the staining conditions of each channel. Then, the unified algorithm and the threshold for each channel were applied to all samples of the slide to standardize the fluorescence level of each marker. Finally, the positively stained cell population was counted and divided by the total cell number to generate the percentage of positive cells. The mean intensity of positive cells was also calculated.

### Data processing

Data on mRNA expression levels and clinical characteristics were downloaded from an integrated The Cancer Genome Atlas (TCGA) Pan-Cancer Clinical Data Resource. The survival curves were plotted by the Kaplan–Meier method and tested by log-rank analysis. Multivariate analysis was used for the Cox proportional hazard model using R language packages. Least absolute shrinkage and selection operator (LASSO) Cox regression was used for gene selection in the TCGA stomach adenocarcinoma (STAD) cohort. The RandomForest algorithm based on python sklearn software was used to construct a model, and receiver operating characteristic (ROC) curve analysis was used to evaluate the predictive accuracy and sensitivity of the therapy prediction model. Based on the data of “primary_therapy_outcome_success”, outcomes were divided into therapy sensitivity (including CR: complete remission, PR: partial remission/response, SD: stable disease) and therapy resistance (PD: progressive disease). To analyze immune cell proportions, formatted data were uploaded to the CIBERSORT web portal (https://cibersort.standord.edu./). The clinical characteristics of patients with different protein expression levels were compared by using a two-tailed chi-square test with SPSS 20 software (SPSS Inc., USA). A *P* value < 0.05 was considered significant.

### Supplementary information

Supplementary Figures and Tables

Original data files

## Data Availability

All datasets analyzed in the study are available from the corresponding authors on reasonable request.
