# Supplementary material for: TAGLN2 induces resistance signature ISGs by activating AKT-YBX1 signal with dual pathways and mediates the IFN-related DNA damage resistance in gastric cancer
Source: Cell Death Dis. 2024 Aug 21;15(8):608. doi: 10.1038/s41419-024-07000-1 (PMC11339399; doi:10.1038/s41419-024-07000-1)
Supplement: Supplementary file 1 — Supplementary Figures and Tables [file 41419_2024_7000_MOESM1_ESM.docx]

**
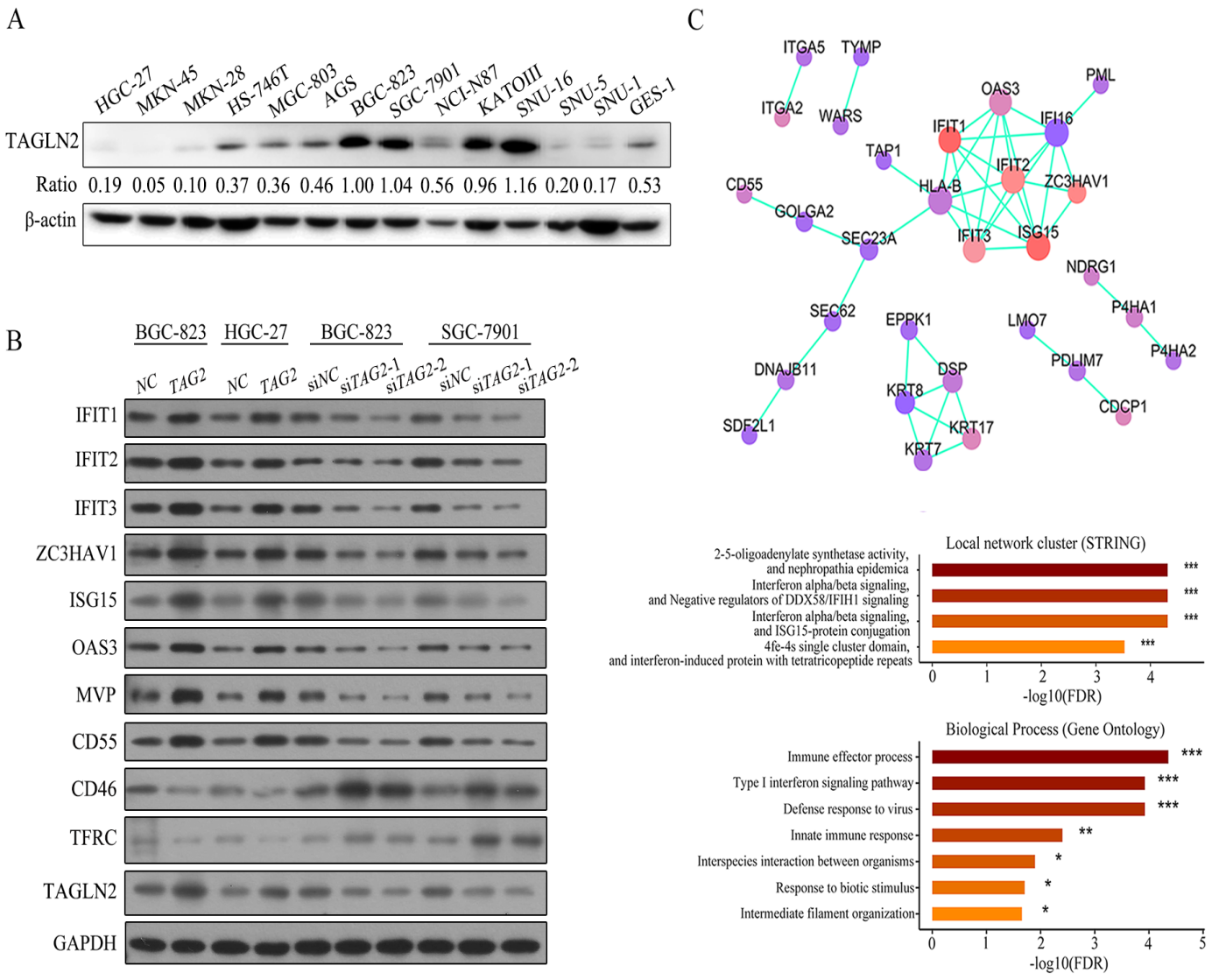
**

**Fig. 1 Expression of TAGLN2 and related molecules detected by Western blot and RNA-seq analysis.**

(A) Expression of TAGLN2 was detected in 13 GC cell lines and the human gastric mucosal cell line GES-1. (B) The protein expression profile in aberrant TAGLN2-expressing cells was validated by Western blot of selected proteins from independent samples of BGC-823, HGC-27 and SGC7901 versus the control cell line. (C) iTRAQ-2DLC‒MS/MS analyses showed that aberrantly upregulated TAGLN2 expression results elevates the expression of interferon-stimulated genes.


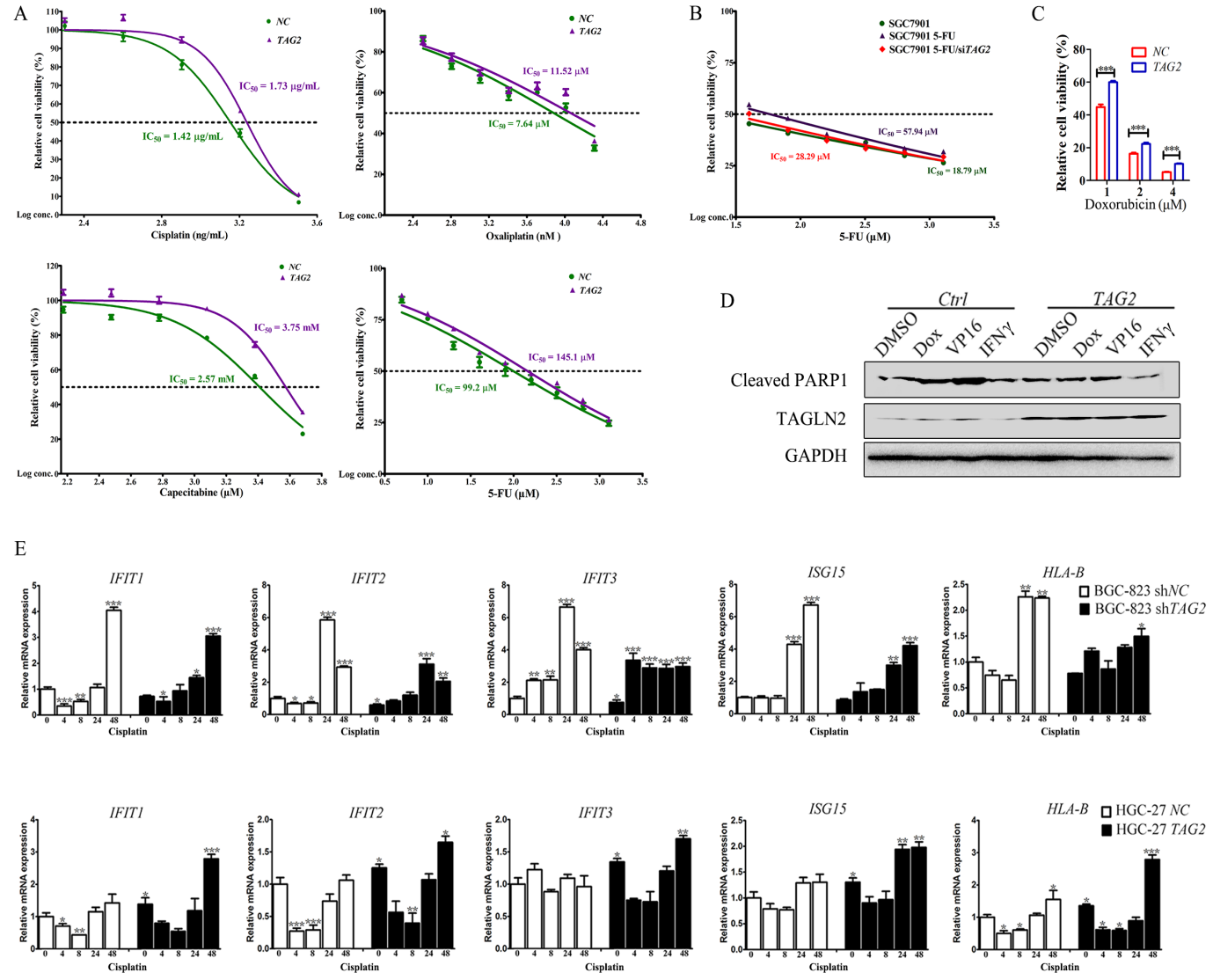


**Fig. 2 Upregulated TAGLN2 expression induced therapy resistance and ISGs upregulation in GC cell lines.** (A) The IC_50_ of Cisplatin, Oxaliplatin, Capecitabine and 5-FU were calculated in HGC-27 cells with TAGLN2 overexpression using Cell Counting Kit-8. (B) The IC_50_ of 5-FU was calculated in drug-resistance cell line SGC-7901/5-FU, without or with *TAGLN2* knockdown. (C) Cell viability of HGC-27 cells overexpressing TAGLN2 was determined after Doxorubicin (Dox) treatment at concentrations of 1, 2, 4 μM for 48 h. (D) The cleavage level of PARP, which is involved in cell apoptosis, was detected by Western blot after Dox, VP16 and IFNγ treatment in HGC-27 cells overexpressing TAGLN2. (E) Expression of the gene panel in BGC-823 cells with *TAGLN2* knockdown or HGC-27 cells with *TAGLN2* overexpression after cisplatin (1 μg/ml) treatment.

**
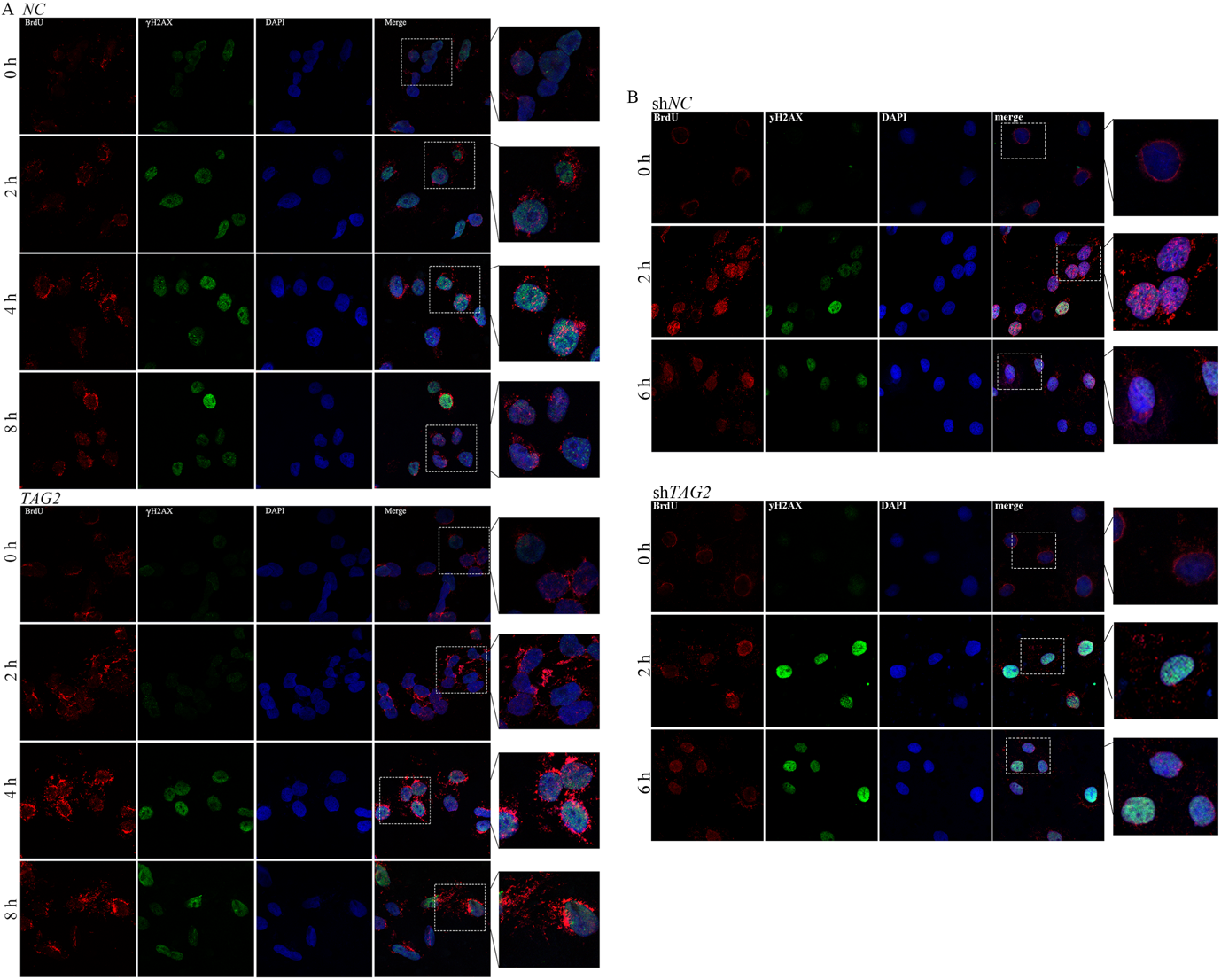
**

**Fig. 3 Depletion of *TAGLN2* markedly reduced cytosolic ssDNA accumulation, as determined by BrdU-γH2AX double labeling.** BGC-823 cells with stable *TAGLN2* knockdown (A) or HGC-27 with stable *TAGLN2* overexpression (B) were prelabeled with 10 μg/ml BrdU for approximately 1.5 cell cycles and subsequently treated with 1 μg/ml cisplatin over the subsequent 2 to 8 h. Samples were incubated with primary antibodies against BrdU (red) and phospho-histone H2AX (green) for 3 h and with secondary antibodies for 1.5 h at room temperature. Slides were mounted with VectaShield antifade mounting medium with DAPI (blue). Images were taken using a 63× objective on a Zeiss LSM780 confocal microscope.

**Table 1 Correlation between TAGLN2 expression and clinicopathologic factors of human gastric cancer by immunohistochemistry analysis**

| Features | Cases | TAGLN2 Expression  (cytoplasm) | | TAGLN2 Expression  (nucleus) | |
| --- | --- | --- | --- | --- | --- |
|  |  | Pearson's R | *P* value | Pearson's R | *P* value |
| Gender |  |  |  |  |  |
| Male | 59 | 0.093 | 0.446 | 0.146 | 0.228 |
| Female | 16 |  |  |  |  |
| Age (years) |  |  |  |  |  |
| ≤ Average | 37 | 0.179 | 0.138 | -0.215 | 0.073 |
| > Average | 37 |  |  |  |  |
| Histology grade |  |  |  |  |  |
| Moderately differentiated | 22 | -0.038 | 0.751 | 0.053 | 0.659 |
| Poorly differentiated | 53 |  |  |  |  |
| Stage |  |  |  |  |  |
| I | 7 | **0.457 ^∗∗^** | **<0.0001** | 0.033 | 0.782 |
| II | 31 |  |  |  |  |
| III | 27 |  |  |  |  |
| IV | 10 |  |  |  |  |
| Tumor Size |  |  |  |  |  |
| Size ≤ 3 cm | 12 | 0.134 | 0.266 | -0.118 | 0.328 |
| 3 < Size ≤5 cm | 31 |  |  |  |  |
| 5 < Size ≤7 cm | 18 |  |  |  |  |
| Size> 7 cm | 14 |  |  |  |  |
| T stage |  |  |  |  |  |
| T1 | 4 | **0.335 ^∗∗^** | **0.005** | 0.111 | 0.363 |
| T2 | 10 |  |  |  |  |
| T3 | 41 |  |  |  |  |
| T4 | 18 |  |  |  |  |
| N stage |  |  |  |  |  |
| N0 | 24 | 0.150 | 0.215 | **-0.237 ^∗^** | **0.049** |
| N1 | 14 |  |  |  |  |
| N2 | 15 |  |  |  |  |
| N3 | 21 |  |  |  |  |
| M stage |  |  |  |  |  |
| M0 | 65 | **0.414 ^∗∗^** | **<0.0001** | 0.110 | 0.363 |
| M1 | 10 |  |  |  |  |

**P* < 0.05, ***P* < 0.01

**Table 2 Differentially expressed proteins or genes related to *TAGLN2* overexpression or downregulation in BGC-823 cells identified by iTRAQ-2DLC-MS/MS and RNA-Seq analysis**

| **NO.** | **Symbol** | **Description** | **Ratio (iTRAQ *TAG2/NC*)/log2 (*TAN2/NC*)/ log2 (*siTAG2/siNC*)** |
| --- | --- | --- | --- |
| 1 | IFIT1 | Interferon-Induced Protein With Tetratricopeptide Repeats 1 | 3.31/2.47 |
| 2 | ISG15 | Interferon-Induced 15 KDa Protein | 3.22/1.87/-2.90 |
| 3 | TAGLN2 | Transgelin 2 | 2.57/1.11/-2.87 |
| 4 | IFIT2 | Interferon Induced Protein With Tetratricopeptide Repeats 2 | 2.31/3.32 |
| 5 | ZC3HAV1 | Zinc Finger CCCH-Type Containing, Antiviral 1 | 2.35/1.90 |
| 6 | MVP | Major Vault Protein | 2.18 |
| 7 | IFIT3 | Interferon Induced Protein With Tetratricopeptide Repeats 3 | 1.90/3.30 |
| 8 | OAS3 | 2'-5'-Oligoadenylate Synthetase 3 | 1.70 |
| 9 | CD55 | Complement Decay-Accelerating Factor | 1.61/-1.04 |
| 10 | ITGA2 | Integrin Subunit Alpha 2 | 1.70/-1.17 |
| 11 | HLA-B | Major Histocompatibility Complex, Class I, B | 1.52/-2.26 |
| 12 | WARS | Tryptophanyl-TRNA Synthetase 1 | 1.44 |
| 13 | ITGA5 | Integrin Subunit Alpha 5 | 1.36 |
| 14 | GOLGA2 | Golgi Autoantigen, Golgin Subfamily A, 2 | 1.33 |
| 15 | PML | Tripartite Motif-Containing Protein 19 | 1.41 |
| 16 | TAP1 | Transporter 1, ATP Binding Cassette Subfamily B Member | 1.27 |
| 17 | SDF2L1 | Stromal Cell Derived Factor 2 Like 1 | 1.28 |
| 18 | TYMP | Thymidine Phosphorylase | 1.32 |
| 19 | SEC23A | SEC23 Homolog A, COPII Coat Complex Component | 1.27 |
| 20 | CTSA | Cathepsin A | 1.24/-1.33 |
| 21 | DHRS7 | Dehydrogenase/Reductase 7 | 1.30 |
| 22 | SPAG9 | Sperm Associated Antigen 9 | 1.25 |
| 23 | LPCAT1 | Lysophosphatidylcholine Acyltransferase 1 | 1.30 |
| 24 | NDUFB1 | NADH:Ubiquinone Oxidoreductase Subunit B1 | 1.25 |
| 25 | AHNAK2 | AHNAK Nucleoprotein 2 | 1.30 |
| 26 | SEC62 | SEC62 Homolog, Preprotein Translocation Factor | 1.27 |
| 27 | MAGED2 | MAGE Family Member D2 | 1.21 |
| 28 | RABGGTB | Rab Geranylgeranyltransferase Subunit Beta | 1.20 |
| 29 | TMCO1 | Transmembrane And Coiled-Coil Domains 1 | 1.23 |
| 30 | SAMM50 | Sorting And Assembly Machinery Component 50 Homolog | 1.23 |
| 31 | IFI16 | Interferon Gamma Inducible Protein 16 | 1.21 |
| 32 | ABCA10 | ATP Binding Cassette Subfamily A Member 10 | 2.58/-5.92 |
| 33 | AIM2 | Absent In Melanoma 2 | 2.86 |
| 34 | ARID5A | AT-Rich Interaction Domain 5A | 1.04 |
| 35 | ARNTL2 | Aryl Hydrocarbon Receptor Nuclear Translocator Like 2 | 1.51/-1.95 |
| 36 | BST1 | Bone Marrow Stromal Cell Antigen 1 | 1.42/-5.73 |
| 37 | CCL22 | C-C Motif Chemokine Ligand 22 | 1.5/-2.11 |
| 38 | CCL26 | C-C Motif Chemokine Ligand 26 | 1.39 |
| 39 | CCL3L3 | C-C Motif Chemokine Ligand 3 Like 3 | 6.25 |
| 40 | CCL5 | C-C Motif Chemokine Ligand 5 | 2.1/-2.67 |
| 41 | CDC42EP2 | CDC42 Effector Protein 2 | 1.26 |
| 42 | CEACAM1 | CEA Cell Adhesion Molecule 1 | 1.44/-1.84 |
| 43 | CX3CL1 | C-X3-C Motif Chemokine Ligand 1 | 3.62 |
| 44 | CXCL11 | C-X-C Motif Chemokine Ligand 11 | 5.30 |
| 45 | CYP3A7 | Cytochrome P450 Family 3 Subfamily A Member 7 | 1.12/-1.99 |
| 46 | DDX58 | RNA Helicase RIG-I | 1.91/-1.54 |
| 47 | DHRS2 | Dehydrogenase/Reductase 2 | 2.14/-1.31 |
| 48 | DHX58 | DExH-Box Helicase 58 | 1.12 |
| 49 | EPO | Erythropoietin | 5.48 |
| 50 | FAM26E | Family With Sequence Similarity 26 Member E | 3.18/-3.11 |
| 51 | FCMR | Fc Mu Receptor | 1.96/-1.49 |
| 52 | GBP5 | Guanylate Binding Protein 5 | 2.10/-6.02 |
| 53 | HEATR4 | HEAT Repeat Containing 4 | 1.13/-1.35 |
| 54 | HERC5 | HECT And RLD Domain Containing E3 Ubiquitin Protein Ligase 5 | 2.02 |
| 55 | HERC6 | HECT And RLD Domain Containing E3 Ubiquitin Protein Ligase Family Member 6 | 1.37/-1.38 |
| 56 | HIST1H2BJ | Histone Cluster 1 H2B Family Member J | 1.14 |
| 57 | HMGA2 | High Mobility Group AT-Hook 2 | 1.18/-1.07 |
| 58 | ICAM1 | Intercellular Adhesion Molecule 1 | 1.29 |
| 59 | IFI44 | Interferon Induced Protein 44 | 2.35/-2.30 |
| 60 | IFI6 | Interferon Alpha Inducible Protein 6 | 1.24/-2.01 |
| 61 | IFIH1 | Interferon Induced With Helicase C Domain 1 | 1.71 |
| 62 | IL18 | Interleukin 18 | 1.82 |
| 63 | IL1R1 | Interleukin 1 Receptor Type 1 | 2.46/-1.79 |
| 64 | IL1RN | Interleukin 1 Receptor Antagonist | 2.03/-5.88 |
| 65 | IL7R | Interleukin 7 Receptor | 1.37/-3.60 |
| 66 | IRAK2 | Interleukin 1 Receptor Associated Kinase 2 | 1.31 |
| 67 | IRGC | Immunity Related GTPase Cinema | 2.36 |
| 68 | ISG20 | Interferon Stimulated Exonuclease Gene 20 | 1.86 |
| 69 | KITLG | Mast Cell Growth Factor | 1.78 |
| 70 | LGALS9 | Galectin 9 | 1.23 |
| 71 | MARCH4 | Membrane Associated Ring-CH-Type Finger 4 | 1.40/-2.45 |
| 72 | MICA | MHC Class I Polypeptide-Related Sequence A | 1.28 |
| 73 | NAV3 | Neuron Navigator 3 | 1.28/-1.17 |
| 74 | NCF2 | Neutrophil Cytosolic Factor 2 | 2.94/-1.77 |
| 75 | OAS2 | 2'-5'-Oligoadenylate Synthetase 2 | 1.84/-1.78 |
| 76 | OASL | 2'-5'-Oligoadenylate Synthetase Like | 3.46/-1.51 |
| 77 | PLXNA4 | Plexin A4 | 1.24/-7.21 |
| 78 | RAB7B | Ras-Related Protein Rab-7 | 3.04 |
| 79 | RBM14 | RNA Binding Motif Protein 14 | 1.07 |
| 80 | RCSD1 | RCSD Domain Containing 1 | 1.41/-3.06 |
| 81 | RSAD2 | Radical S-Adenosyl Methionine Domain Containing 2 | 2.40/-3.16 |
| 82 | SOCS1 | Suppressor Of Cytokine Signaling 1 | 2.12 |
| 83 | SPINK6 | Serine Peptidase Inhibitor Kazal Type 6 | 1.73/-2.40 |
| 84 | SPOCK1 | SPARC (Osteonectin), Cwcv And Kazal Like Domains Proteoglycan 1 | 1.58/-2.55 |
| 85 | TEPP | Testis, Prostate And Placenta Expressed | 2.25/-3.34 |
| 86 | TFPI2 | Tissue Factor Pathway Inhibitor 2 | 1.08/-1.21 |
| 87 | TFRC | Transferrin Receptor | 1.29 |
| 88 | TMEM173 | Stimulator Of Interferon Response CGAMP Interactor 1 | 1.51 |
| 89 | UCA1 | Urothelial Cancer Associated 1 | 1.47/-1.11 |
| 90 | ULBP3 | UL16 Binding Protein 3 | 1.68 |
| 91 | YBX1 | Y-Box Binding Protein 1 | 2.08/-1.92 |
| 92 | ZBTB32 | Zinc Finger And BTB Domain Containing 32 | 2.15/-3.82 |

**Table 3 Potential TAGLN2-binding proteins identified using Co-IP followed by LC-MS/MS**

| **NO.** | **Symbol** | **Score** | **Mass** | **Matches** | **Sequences** | **emPAI** | **Protein description** |
| --- | --- | --- | --- | --- | --- | --- | --- |
| 1 | TAGLN2 | 366 | 22548 | 19 (15) | 8 (7) | 3.6 | Transgelin-2 |
| 2 | YBX1 | 233 | 42333 | 9 (7) | 8 (6) | 0.57 | Nuclease-sensitive element-binding protein 1 |
| 3 | HIST1H1D | 201 | 22336 | 24 (6) | 7 (5) | 1.32 | Histone H1.3 |
| 4 | Actin | 156 | 40116 | 12 (7) | 8 (6) | 0.74 | cDNA FLJ57283, highly similar to Actin, cytoplasmic 2 |
| 5 | HSP90AB1 | 94 | 82518 | 6 (1) | 5 (1) | 0.04 | highly similar to Heat shock protein HSP 90-beta |
| 6 | ANXA2 | 85 | 38808 | 2 (1) | 2 (1) | 0.09 | Annexin A2 |

**Table 4 Clinical data related to 90 gastric cancer specimens on the tissue microarray chip**

| **NO.** | **Gender** | **Age** | **Histological grade** | **Size (cm)** | **Number of lymph nodes** | **Number of positive lymph nodes** | **T** | **N** | **M** | **Stage** | **PDL1^+^ (%)** | **CD8^+^ (%)** | **Survival time** |
| --- | --- | --- | --- | --- | --- | --- | --- | --- | --- | --- | --- | --- | --- |
| 1 | F | 52 | III-IV | 7×5×2 | 19 | 16 | T4a | N3b | M0 | 3C | 50 | 10 | 3 |
| 2 | F | 74 | III-IV | - | 38 | 34 | T3 | N3b | M0 | 3B | 0 | 5 | 3 |
| 3 | M | 67 | II-III | 10×9×1.6 | 12 | 10 | T3 | N3a | M0 | 3B | 30 | 10 | 1 |
| 4 | M | 72 | III | 7×6×1.5 | 5 | 4 | T3 | N2 | M0 | 3A | 30 | 5 | 2 |
| 5 | M | 73 | III | 9×5×1.5 | 16 | 16 | T4a | N3b | M0 | 3C | 50 | 15 | 3 |
| 6 | F | 42 | II-III | 7×7×1 | 14 | 6 | T4b | N2 | M0 | 3C | 5 | 5 | 8 |
| 7 | M | 61 | III-IV | 3×3×1 | 10 | 0 | T3 | N0 | M0 | 2A | 10 | 15 | 88 |
| 8 | M | 56 | III | 3×2×1 | 13 | 7 | T3 | N3a | M0 | 3B | - | 5 | 88 |
| 9 | F | 54 | III-IV | 11×10×1 | 7 | 4 | T4b | N2 | M0 | 3C | 5 | 10 | 5 |
| 10 | M | 60 | III | 1.5×1×1 | 18 | 2 | T3 | N1 | M0 | 2B | 1 | 20 | 42 |
| 11 | M | 62 | II | 4×2.8×0.3 | 17 | 0 | T1b | N0 | M0 | 1A | 1 | 5 | 88 |
| 12 | F | 79 | II-III | 5.5×4×1 | 8 | 0 | T3 | N0 | M0 | 2A | 5 | 5 | 17 |
| 13 | F | 69 | I-II | 5×4×2 | 12 | 2 | T3 | N1 | M0 | 2B | 30 | 5 | 88 |
| 14 | M | 57 | III | 5×4×1 | 22 | 11 | T3 | N3a | M0 | 3B | 1 | 15 | 77 |
| 15 | M | 68 | III | 9×6×1 | 14 | 11 | T4a | N3a | M0 | 3C | 10 | 15 | 8 |
| 16 | M | 53 | III | 4.5×3.5×1 | 6 | 2 | T3 | N1 | M0 | 2B | 1 | 5 | 17 |
| 17 | M | 71 | II-III | 9×5.5×1 | 22 | 4 | T4a | N2 | M0 | 3B | 35 | 20 | 10 |
| 18 | F | 68 | II-III | 4×4×1.5 | 1 | 1 | T4a | N1 | M0 | 3A | 5 | 5 | 5 |
| 19 | M | 61 | III | 13×5.5×2 | 10 | 10 | T4a | N3a | M0 | 3C | 5 | 2 | 0 |
| 20 | F | 68 | II | 7.5×5.5×2 | 7 | 1 | T3 | N1 | M0 | 2B | 5 | <1 | 11 |
| 21 | M | 71 | III | 7×4×1.5 | 8 | 3 | T3 | N2 | M0 | 3A | 1 | 1 | 44 |
| 22 | F | 65 | III | 4×2.5×1.5 | 12 | 0 | T3 | N0 | M0 | 2A | 30 | 20 | 88 |
| 23 | M | 72 | III | 4.5×4×1.5 | 10 | 6 | T2 | N2 | M1 | 4 | 5 | 2 | 6 |
| 24 | M | 49 | III-IV | 5×4×1.5 | 12 | 0 | T3 | N0 | M0 | 2A | 5 | 10 | 1 |
| 25 | M | 50 | II-III | 7.5×3.5×1.5 | 20 | 4 | T4a | N2 | M0 | 3B | 20 | 10 | 88 |
| 26 | F | 52 | III-IV | 6×3×1.5 | 21 | 19 | T3 | N3b | M0 | 3B | 5 | 5 | 16 |
| 27 | M | 78 | III | 15×9×1.5 | 17 | 16 | T3 | N3b | M0 | 3B | 2 | 5 | 8 |
| 28 | M | 67 | III | 10×10×1.5 | 9 | 0 | T3 | N0 | M0 | 2A | 60 | 5 | 88 |
| 29 | M | 75 | II-III | 6×4×1 | 13 | 13 | T4b | N3a | M0 | 3C | 20 | 15 | 0 |
| 30 | M | 65 | III | 6×4×1 | - | 0 | T3 | N0 | M0 | 2A | - | 30 | 3 |
| 31 | M | 75 | III | 6×6×2 | 34 | 34 | T4a | N3b | M0 | 3C | 80 | 5 | 7 |
| 32 | M | 78 | III | 8×8×5 | 7 | 0 | T3 | N0 | M0 | 2A | 50 | 15 | 87 |
| 33 | M | 51 | III | 7×4×1.5 | 4 | 4 | T3 | N2 | M0 | 3A | 25 | 5 | 10 |
| 34 | M | 74 | II-III | 2.8×2×1 | 7 | 3 | T4a | N2 | M0 | 3B | 15 | 10 | 67 |
| 35 | F | 67 | III | 5×4×2 | 18 | 10 | T3 | N3a | M0 | 3B | 5 | 5 | 4 |
| 36 | M | 62 | III | 7.5×5×2.5 | - | 0 | T3 | N0 | M0 | 2A | 1 | 10 | 12 |
| 37 | M | 66 | III | 7×5×1 | 13 | 13 | T4a | N3a | M0 | 3C | 10 | 10 | 2 |
| 38 | M | 72 | III-IV | 4×2.5×1 | 13 | 0 | T3 | N0 | M0 | 2A | 5 | 15 | 87 |
| 39 | M | 67 | II-III | 3.5×3×1.5 | 6 | 0 | T3 | N0 | M0 | 2A | 5 | 10 | 87 |
| 40 | M | 73 | II | 3.5×3.5×1 | 9 | 3 | T3 | N2 | M0 | 3A | 10 | - | 26 |
| 41 | F | 76 | II-III | 2.5×2×1 | 2 | 2 | T3 | N1 | M0 | 2B | 10 | 20 | 14 |
| 42 | F | 80 | III | 7×4.5×1.5 | 8 | 0 | T3 | N0 | M0 | 2A | 20 | 10 | 87 |
| 43 | M | 59 | III | 3×2×1.5 | 6 | 4 | T4a | N2 | M0 | 3B | 10 | 5 | 54 |
| 44 | M | 58 | II | 7×4×1.5 | 10 | 5 | T3 | N2 | M0 | 3A | 15 | 2 | 17 |
| 45 | M | 59 | II-III | 10×7×1.5 | 7 | 4 | T3 | N2 | M0 | 3A | 1 | 5 | 14 |
| 46 | M | 74 | II | 5×4×1 | 8 | 0 | T4a | N0 | M0 | 2B | 5 | 10 | 86 |
| 47 | M | 58 | II-III | 3×2×1.5 | 9 | 0 | T2 | N0 | M0 | 1B | 5 | 5 | 86 |
| 48 | F | 51 | III | 3.5×2×1 | 21 | 0 | T3 | N0 | M0 | 2A | 5 | 10 | 86 |
| 49 | F | 31 | III-IV | 2.5×2.5×1 | 10 | 0 | T2 | N0 | M0 | 1B | 5 | 5 | 86 |
| 50 | F | 76 | III | 8×7×1 | 16 | 8 | T3 | N3a | M0 | 3B | 15 | 15 | 30 |
| 51 | M | 64 | II | 7×5×0.5 | 17 | 2 | T2 | N1 | M0 | 2A | 1 | 10 | 86 |
| 52 | M | 52 | III-IV | 4×3×2;2×2;2×1.5 | 21 | 1 | T3 | N1 | M0 | 2B | 0 | 15 | 22 |
| 53 | M | 54 | III | 3×3×1 | 20 | 7 | T3 | N3a | M0 | 3B | 1 | 10 | 19 |
| 54 | M | 58 | III | 6×5×1.5 | 11 | 0 | T3 | N0 | M0 | 2A | 30 | 15 | 85 |
| 55 | M | 83 | II | 8×5×1 | 10 | 8 | T3 | N3a | M0 | 3B | 0 | 1 | 5 |
| 56 | M | 65 | II | 9×8×2 | 14 | 0 | T3 | N0 | M0 | 2A | 0 | 2 | 85 |
| 57 | M | 63 | III | 15×11×1 | 19 | 18 | T3 | N3b | M0 | 3B | 5 | 5 | 15 |
| 58 | M | 67 | III | 3.5×1.5×1 | 11 | 0 | T3 | N0 | M0 | 2A | 40 | 10 | 85 |
| 59 | F | 65 | III | 5×4×1 | 22 | 22 | T3 | N3b | M0 | 3B | 5 | 5 | 28 |
| 60 | M | 68 | II-III | 4.5×3.5×1.5 | 12 | 0 | T2 | N0 | M0 | 1B | 10 | 5 | 85 |
| 61 | M | 80 | III | 7.5×4.5×1.5 | 13 | 12 | T3 | N3a | M0 | 3B | 20 | 5 | 10 |
| 62 | M | 54 | III-IV | 5.5×3×1.5 | 10 | 0 | T3 | N0 | M0 | 2A | 50 | 15 | 85 |
| 63 | M | 49 | III-IV | 4×3×2 | 16 | 8 | T3 | N3a | M0 | 3B | 10 | 10 | 85 |
| 64 | M | 54 | II | 10×9×2 | 10 | 3 | T3 | N2 | M0 | 3A | 1 | 5 | 15 |
| 65 | M | 72 | III | 9×6×2 | 18 | 1 | T3 | N1 | M0 | 2B | 50 | 30 | 22 |
| 66 | M | 72 | III | 5.5×5×1 | 29 | 13 | T3 | N3a | M0 | 3B | 1 | 1 | 84 |
| 67 | F | 46 | I-II | 4×2.5×0.5 | 10 | 0 | T1b | N0 | M0 | 1A | 5 | 5 | 84 |
| 68 | F | 74 | III-IV | 27×12×1.3 | 18 | 9 | T3 | N3a | M0 | 3B | 5 | 10 | 8 |
| 69 | F | 60 | III | 9×6×1 | 9 | 4 | T4a | N2 | M0 | 3B | 5 | 5 | 17 |
| 70 | M | 68 | III | 5.5×5×1.5 | 16 | 3 | T3 | N2 | M0 | 3A | 1 | 5 | 18 |
| 71 | F | 68 | III-IV | 20×13×1.5 | 21 | 0 | T3 | N0 | M0 | 2A | 1 | 10 | 54 |
| 72 | M | 61 | II | 7×5×1.5 | 21 | 3 | T3 | N2 | M0 | 3A | 40 | 40 | 50 |
| 73 | F | 61 | III | 7×6.5×2 | 9 | 5 | T3 | N2 | M0 | 3A | 10 | - | 6 |
| 74 | F | 77 | II-III | 5×5×1 | 21 | 4 | T4a | N2 | M0 | 3B | 20 | 10 | 84 |
| 75 | M | 68 | III | 3×3×1 | 13 | 0 | T3 | N0 | M0 | 2A | 30 | 35 | 84 |
| 76 | M | 59 | II | 4.5×3×2 | 16 | 13 | T3 | N3a | M0 | 3B | 5 | 5 | 38 |
| 77 | M | 56 | III-IV | 5×3×1 | 15 | 13 | T3 | N3a | M0 | 3B | 1 | 10 | 84 |
| 78 | M | 57 | II-III | 5×3×0.8 | 15 | 0 | T2 | N0 | M0 | 1B | 20 | 25 | 84 |
| 79 | F | 78 | II-III | 5.5×4×1.3 | 10 | 3 | T4a | N2 | M0 | 3B | 40 | 35 | 84 |
| 80 | F | 75 | III | 4×2.5×1.5 | 12 | 11 | T3 | N3a | M0 | 3B | 5 | 20 | 24 |
| 81 | M | 76 | II-III | 3.5×2.5×1.5 | 27 | 24 | T4a | N3b | M0 | 3C | 1 | 5 | 0 |
| 82 | M | 77 | III | 4×3×1 | 10 | 3 | T2 | N2 | M0 | 2B | 15 | 30 | 22 |
| 83 | M | 57 | II-III | 4×3×1 | 8 | 8 | T3 | N3a | M0 | 3B | 10 | 5 | 15 |
| 84 | M | 68 | II | 5×4×1.5 | 14 | 4 | T3 | N2 | M0 | 3A | 20 | 10 | 13 |
| 85 | F | 52 | III | 2.5×2×1 | 21 | 17 | T4b | N3b | M0 | 3C | 70 | 20 | 27 |
| 86 | F | 52 | II | 6×5×2 | 13 | 2 | T3 | N1 | M0 | 2B | 35 | 10 | 83 |
| 87 | F | 82 | III | 5×5×1.5 | 8 | 3 | T3 | N2 | M0 | 3A | 1 | 5% | 7 |
| 88 | F | 51 | III | 12×11×1.5 | 26 | 20 | T3 | N3b | M0 | 3B | 5 | 25 | 24 |
| 89 | M | 57 | III | 4×4×1 | 10 | 3 | T4a | N2 | M0 | 3B | 10 | 5 | 83 |
| 90 | M | 69 | II | 8×7×2.5 | 18 | 0 | T3 | N0 | M0 | 2A | 5 | 2 | 83 |

**Table 5 List of Primers for plasmids construction**

| **Gene** | **Foward Primer 5’—3’** | **Revers Primer 5’—3’** |
| --- | --- | --- |
| *TAGLN2* | CCCAAGCTTGCCACCATGGCCAACAGGGGACCTGC | ATAAGAATGCGGCCGCTCAGAGGATCTGGCGTGGCATCCCGTAG |
| *YBX1* | CGCGGATCCGCCACCATGAGCAGCGAGGCCGAG | CCGCTCGAGGCGTCTGCGTCGGTAATTGAAGTTGC |
| *SP1* | CGGGGTACCGCCACCATGAGCGACCAAGATCACTCCAT | CCGCTCGAGTCAGAAGCCATTGCCACTGATATTAATGGA |
| *SOX9* | CGGGGTACCGCCACCATGAATCTCCTGGACCCCTTCAT | CCGCTCGAGTCAAGGTCGAGTGAGCTGTGTGTAGACGG |
| *c-Myc* | CGCGGATCCGCCACCATGCTGGATTTTTTTCGGGTAGTGGA | CCGCTCGAGTTACGCACAAGAGTTCCGTAGCTGTTCAAGTT |

**Table 6 List of si*RNA* target sequences**

| **Gene** |  | **Sense Sequence 5’-3’** | **Antisense** |
| --- | --- | --- | --- |
| *TAGLN2* | #1 | CCAACUGGUUCCCUAAGAAdTdT | UUCUUAGGGAACCAGUUGGdTdT |
|  | #2 | GGCAUUAACACCACUGACAdTdT | UGUCAGUGGUGUUAAUGCCdTdT |
|  | #3 | CCAACUGGCCUCUUCCUUUdTdT | AAAGGAAGAGGCCAGUUGGdTdT |
| *YBX1* | #1 | CCUAUGGGCGUCGACCACAdTdT | UGUGGUCGACGCCCAUAGGdTdT |
| *c-Myc* | #1 | GCUUCACCAACAGGAACUATT | UAGUUCCUGUUGGUGAAGCTT |
|  | #2 | CCUGAGACAGAUCAGCAACAA | UUGUUGCUGAUCUGUCUCAGG |
| *SOX9* | #1 | CGCUCACAGUACGACUACATT | UGUAGUCGUACUGUGAGCGTT |
|  | #2 | GCAGCGACGUCAUCUCCAATT | UUGGAGAUGACGUCGCUGCTT |
| *NC* | #1 | UUCUCCGAACGUGUCACGUTT | ACGUGACACGUUCGGAGAATT |
| *cGAS* | #1 | CAGAAUUCAACUAGAAGAAdTdT | UUCUUCUAGUUGAAUUCUGdTdT |
|  | #2 | GCUGUAACACUUCUUAUUAdTdT | UAAUAAGAAGUGUUACAGCdTdT |
|  | #3 | CUAUUCUCUAGCAACUUAAdTdT | UUAAGUUGCUAGAGAAUAGdTdT |
| *STING* | #1 | GCAUUACAACAACCUGCUAdTdT | UAGCAGGUUGUUGUAAUGCdTdT |
|  | #2 | GGUCAUAUUACAUCGGAUAdTdT | UAUCCGAUGUAAUAUGACCdTdT |
|  | #3 | GCACCUGUGUCCUGGAGUAdTdT | UACUCCAGGACACAGGUGCdTdT |
| *IRF3* | #1 | GGACCAAGAGGCUCGUGAUdTdT | AUCACGAGCCUCUUGGUCCdTdT |
|  | #2 | CUCACCUCCGACCAGUACAdTdT | UGUACUGGUCGGAGGUGAGdTdT |
|  | #3 | CCUCAGGGCCUUGGUAGAAdTdT | UUCUACCAAGGCCCUGAGGdTdT |
| *IFNAR* | #1 | CAAUUGCUACAGUAGAAGAdTdT | UCUUCUACUGUAGCAAUUGdTdT |
|  | #2 | GAAGCUGAAGAUAAGGCAAdTdT | UUGCCUUAUCUUCAGCUUCdTdT |
|  | #3 | GUUAAAGCAGCACUACUUAdTdT | UAAGUAGUGCUGCUUUAACdTdT |

**Table 7 List of Primers for RT‒qPCR**

| **Gene** | **Foward Primer 5’—3’** | **Revers Primer 5’—3’** |
| --- | --- | --- |
| *TAGLN2* | AGCTGAGCGCTATGGCATTA | CAGAGAAGAGCCCATCATCTC |
| *IFIT1* | GGTATGCGATCTCTGCCTAT | GTTGGCTAGAGCTTCTTCAATG |
| *IFIT2* | GAAAGCCCCAGGTGTAACAG | ATGCAGGTAGGCATTGTTTG |
| *IFIT3* | CTCCTTGCCAAACAGATGTC | CCTTGTAGCAGCACCCAAT |
| *IFI16* | ATGAGCCCAAAGAGCAGAAG | ACCCATTGCGGCAAACATA |
| *ISG15* | ACAAATGCGACGAACCTCTGA | AGGCGCAGATTCATGAACAC |
| *HLA-B* | CTGCTGTGATGTGTAGGAGGAAG | GCTGTGAGAGACACATCAGAGC |
| *OAS3* | GGCAGTTCGAGGTCAAGTTT | GCACATCAAAGTCCACACTCT |
| *WARS* | TGTGATGTGGACGTGTCTTTC | GGCTGCAGAACCTCTATGAG |
| *TAP1* | AGTGGTCTGTTGACTCCCTTA | GTGAATGTCAGCCCCTGTAG |
| *MVP* | GTATGTGCCATCTGCCAAAG | CATGTAGGTGCTTCCAATCA |
| *STAT1* | ATTACAAAGTCATGGCTGCT | ATATCCAGTTCCTTTAGGGC |
| *YBX1* | CCTAGAGAGGACGGCAATGA | CGTCTGCGTCGGTAATTGAA |

**Table 8 List of Primers for ChIP-qPCR**

| **Gene** | **Foward Primer 5’—3’** | **Revers Primer 5’—3’** |
| --- | --- | --- |
| YBX1 ChIP1 for TAGLN2 binding | TGTGGCTCATGAGCTAAGAATACAA | TTTTTCTGTGAGAGCCAAATAGTTA |
| YBX1 ChIP2 for TAGLN2 binding | GGAGTATTTACCGTGTTGGCTGTTT | CTGCAACAGCCACGTGATAGAGGGA |
| YBX1 ChIP3 for TAGLN2 binding | TGGAAGTCACGTTCCTTCTGCCCGT | GTACTGTCAATCTGCCCACCTGACC |
| YBX1 ChIP1 for c-Myc binding | TAGTTCTGGATTGAGCCCGTG | TCACCATCTTGGCCAGGCTG |
| YBX1 ChIP2 for c-Myc binding | GTAGTTACAAGCACCTGGAAG | TATCTGATCATGACACTGTCTTAC |
| YBX1 ChIP3 for c-Myc binding | GAAGGCAGAAACTGACTCGCG | ACCACAAATCAGATTTAATTC |
| YBX1 ChIP4 for c-Myc binding | CAGCCGAGACACAACCCTGAAC | CTAGAATTAGAGTAGTCGGCC |
| YBX1 ChIP1 for SOX9 binding | TAGTTCTGGATTGAGCCCGTG | TCACCATCTTGGCCAGGCTG |
| YBX1 ChIP2 for SOX9 binding | ACAGGCCTAGACGGCATTGG | TGGGTTGCAGAGGTGGGCAG |
| YBX1 ChIP3 for SOX9 binding | CGGGTACTCTATGGTTTTCGTG | GGACAGGCGGGATAAGCCCTAC |

**Table 9 List of Primers for Dual luciferase assay**

| **Gene** | **MlulF: 5’—3’** | **XhoIR:5’—3’** |
| --- | --- | --- |
| YBX1-1 (-2000bp~-1bp) | cgacgcgtGTAGTTACAAGCACCTGGAAGGTTGGACAG | ccgctcgagGGCGGGACAGGCGGGATAAGCCCTACGAGC |
| YBX1-2 (-2000bp~-1001bp) | cgacgcgtGTAGTTACAAGCACCTGGAAGGTTGGACAG | ccgctcgagAATTTAAACAGCCAACACGGTAAATACTCC |
| YBX1-3 (-1000bp~-1bp) | cgacgcgtTCTGCCTCCATCAGAAGGCAGAAACTGACT | cgacgcgtTCTGCCTCCATCAGAAGGCAGAAACTGACT |
| YBX1-4 (-1000bp~-501bp) | cgacgcgtTCTGCCTCCATCAGAAGGCAGAAACTGACT | ccgctcgagTTCTTTAATGGCCGTGTAGTCGCGGCACAG |
| YBX1-5 (-500bp~-1bp) | cgacgcgtAAGACGACTCTATGCCCGCCGTAATGTTCT | CcgctcgagGGCGGGACAGGCGGGATAAGCCCTACGAGC |
| YBX1-6 (-500~-166bp) | cgacgcgtAAGACGACTCTATGCCCGCCGTAAT | ccgctcgagTACAACCGTTCCTGCCTCCGCCGGC |
| YBX1-7 (-417~-83bp) | cgacgcgtGGGGGGCTAAGGCGTCTTCGAGCCCCCT | ccgctcgagGACCCCAATCCCTAGCTTCTATTGGCTA |
| YBX1-8 (-334~-1bp) | cgacgcgtTTCAGCCGAGACACAACCCTGAACGTG | ccgctcgagGGCGGGACAGGCGGGATAAGCCCTACG |
