# Supplementary material for: TAGLN2 induces resistance signature ISGs by activating AKT-YBX1 signal with dual pathways and mediates the IFN-related DNA damage resistance in gastric cancer
Source: Cell Death Dis. 2024 Aug 21;15(8):608. doi: 10.1038/s41419-024-07000-1 (PMC11339399; doi:10.1038/s41419-024-07000-1)

Figure 3E

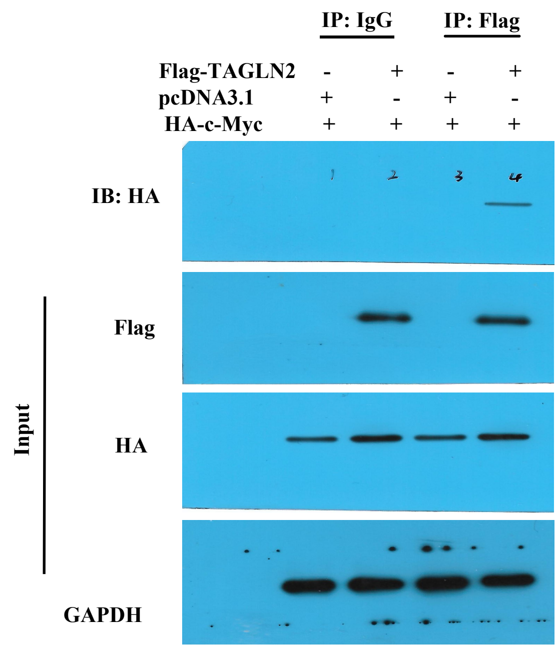

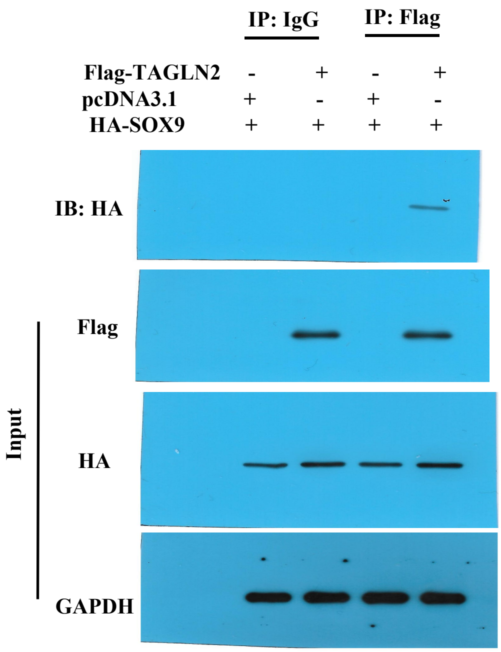

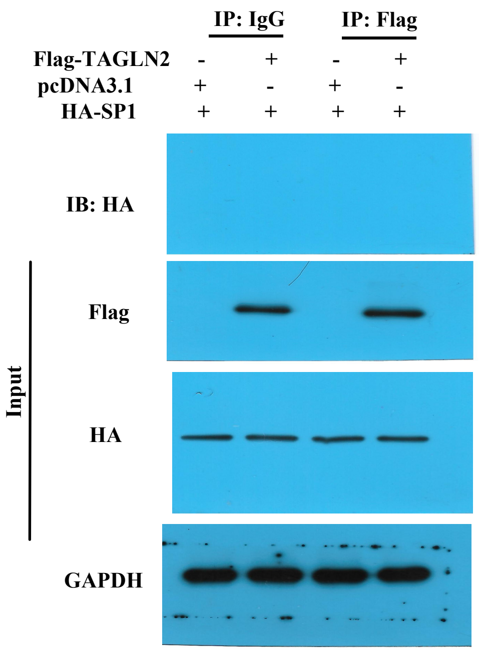

Figure 4G

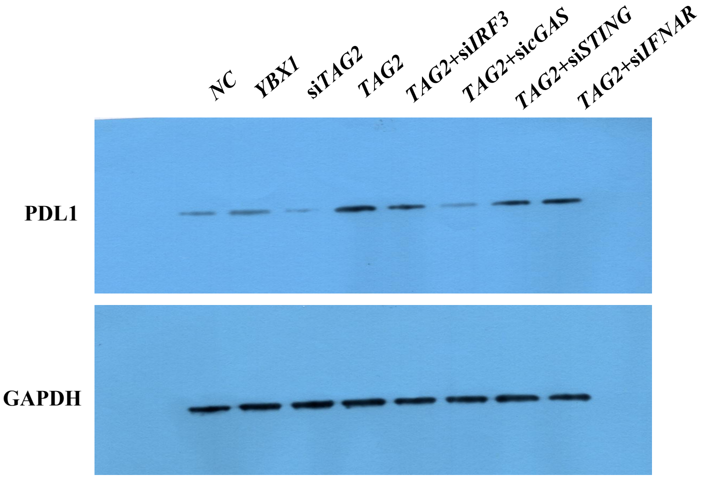

Figure 5A

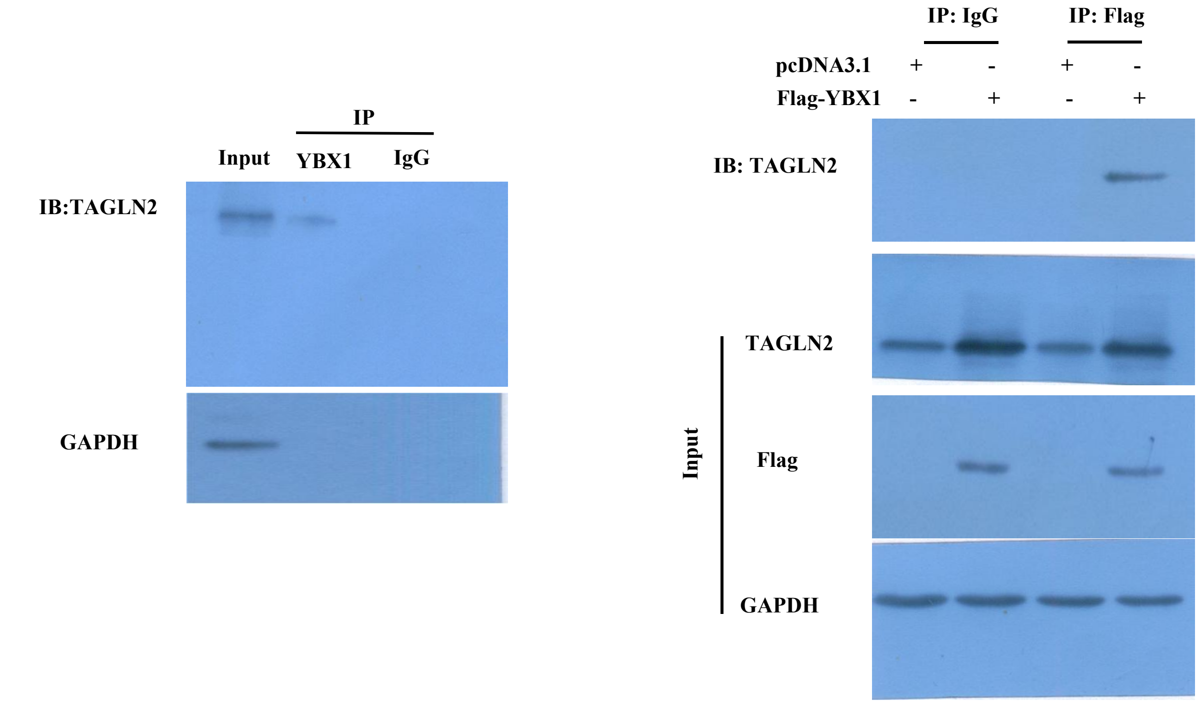

Figure 5B

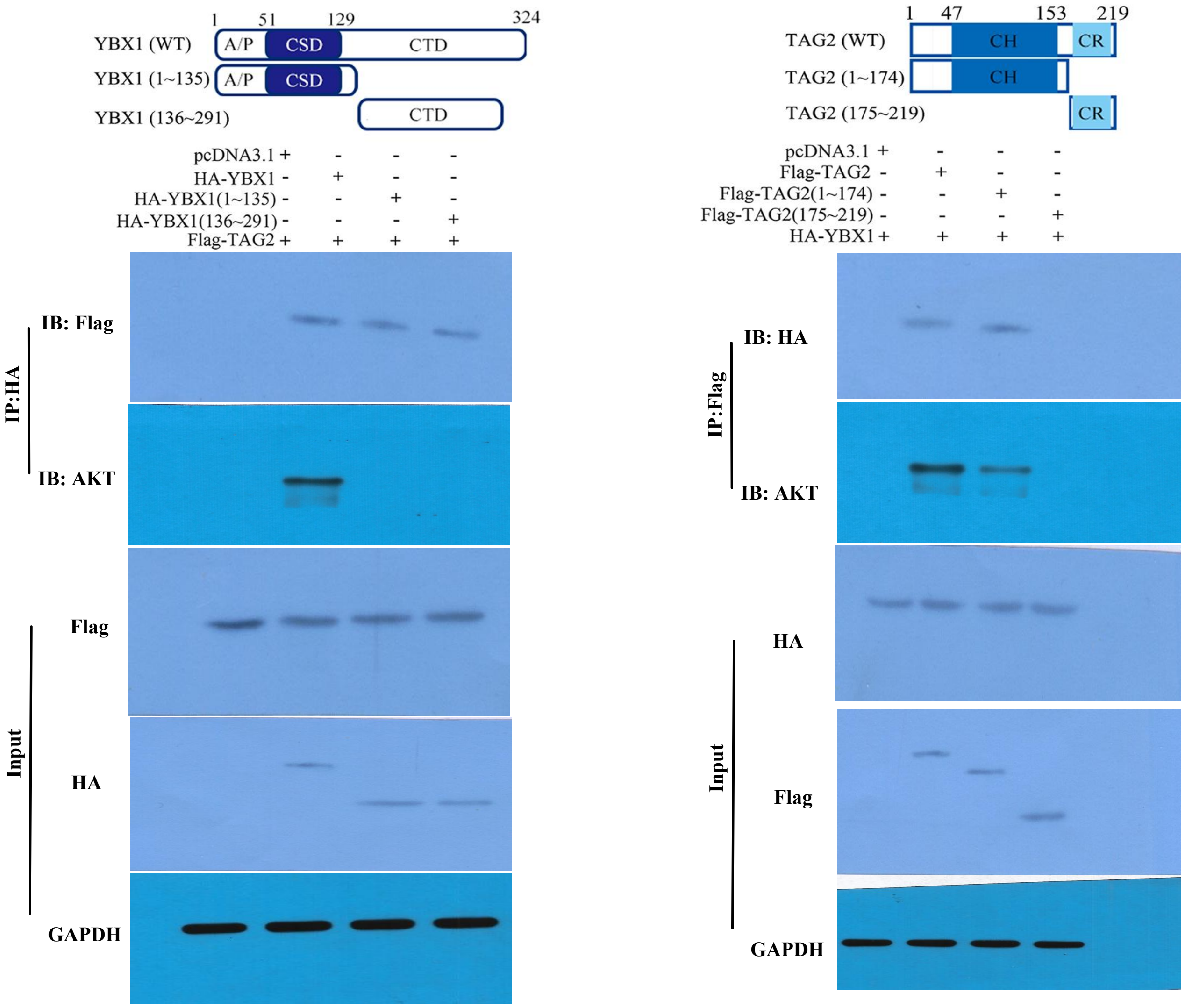

Figure 5B

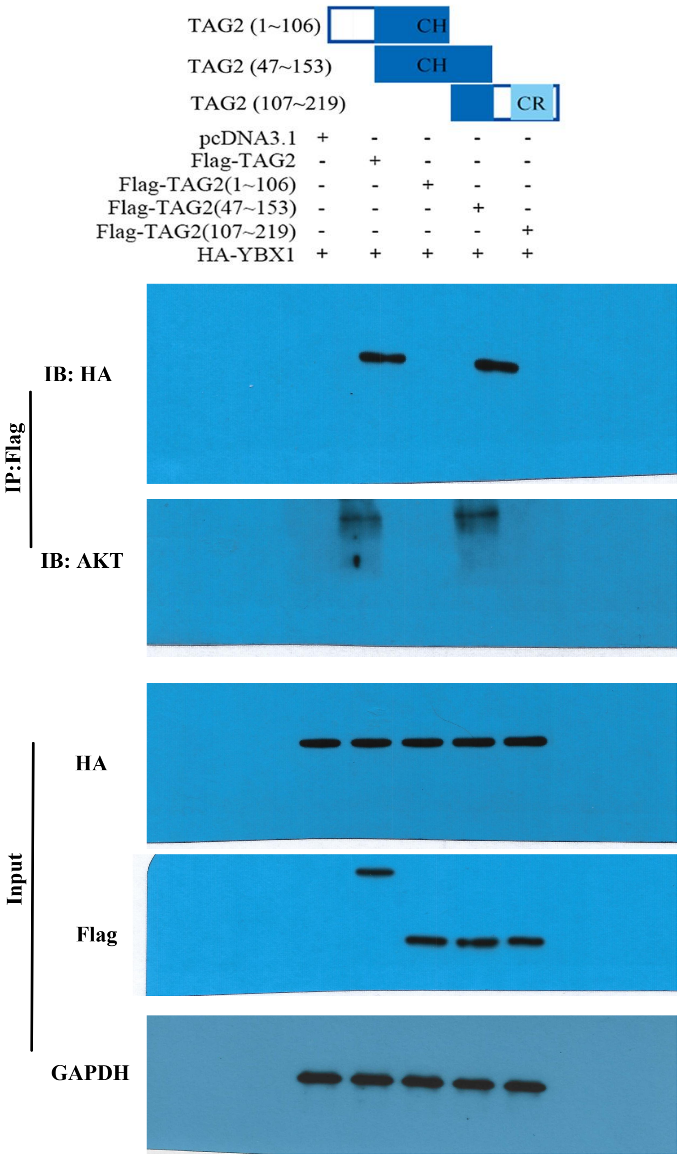

TAG2 (1~106)
CH
TAG2 (47~153)
CH
TAG2 (107~219)
CR
pcDNA3.1 + - - - -
Flag-TAG2 - + - - -
Flag-TAG2(1~106) - - + - -
Flag-TAG2(47~153) - - - + -
Flag-TAG2(107~219) - - - - +
HA-YBX1 + + + + +
IB: HA
IP:Flag
IB: AKT
HA
Input
Flag
GAPDH

Figure 5C

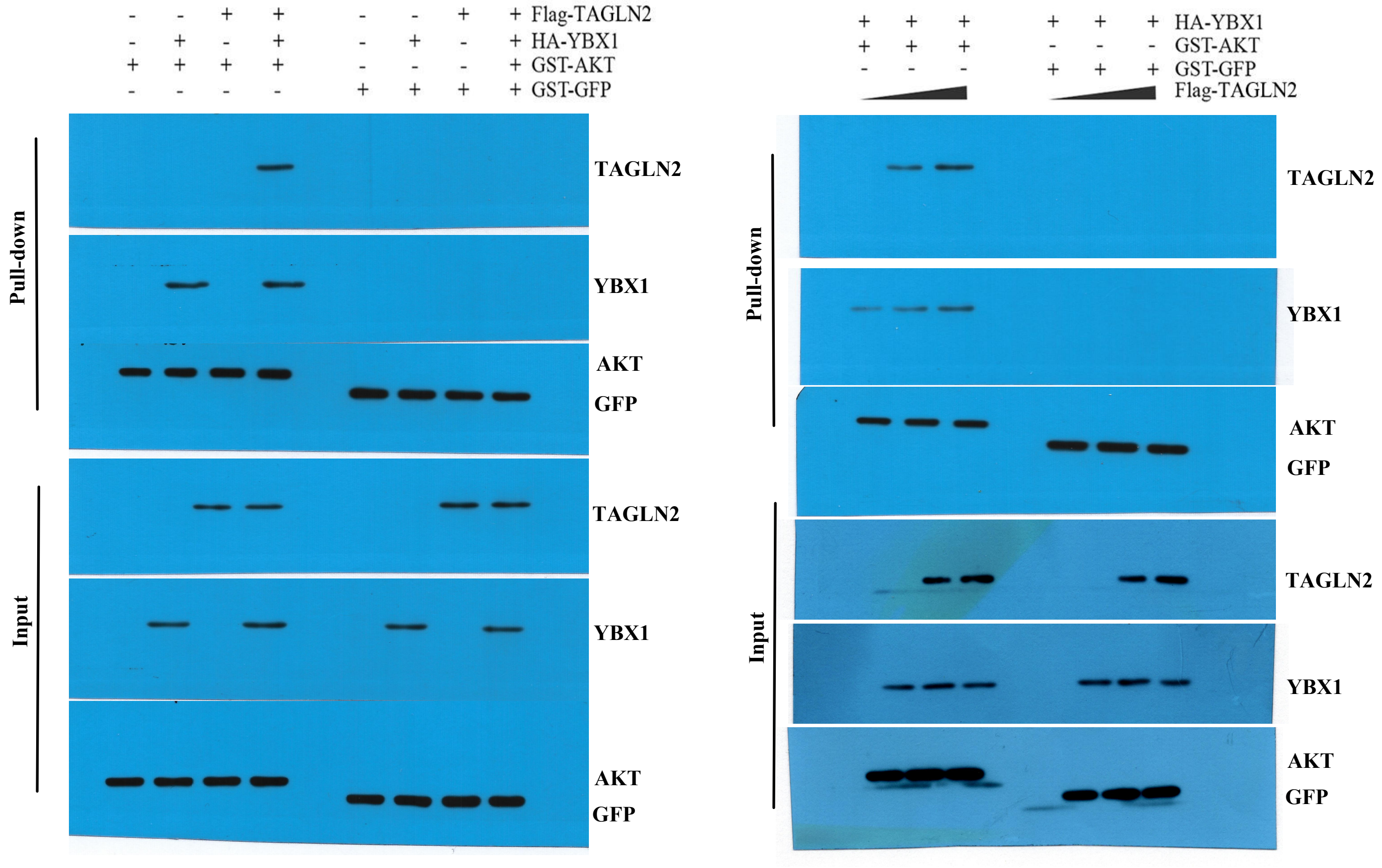

Figure 5D

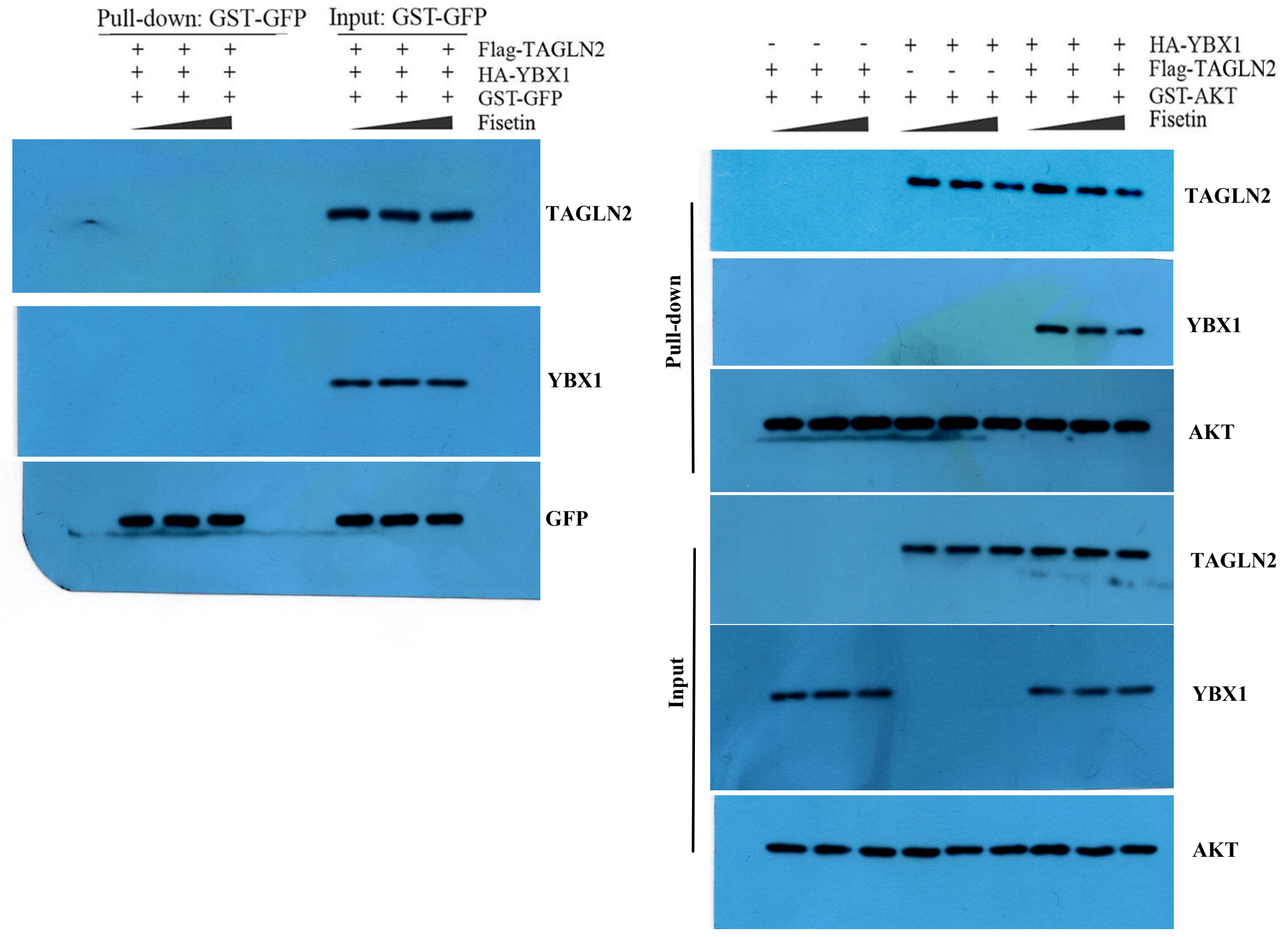

Figure 5E

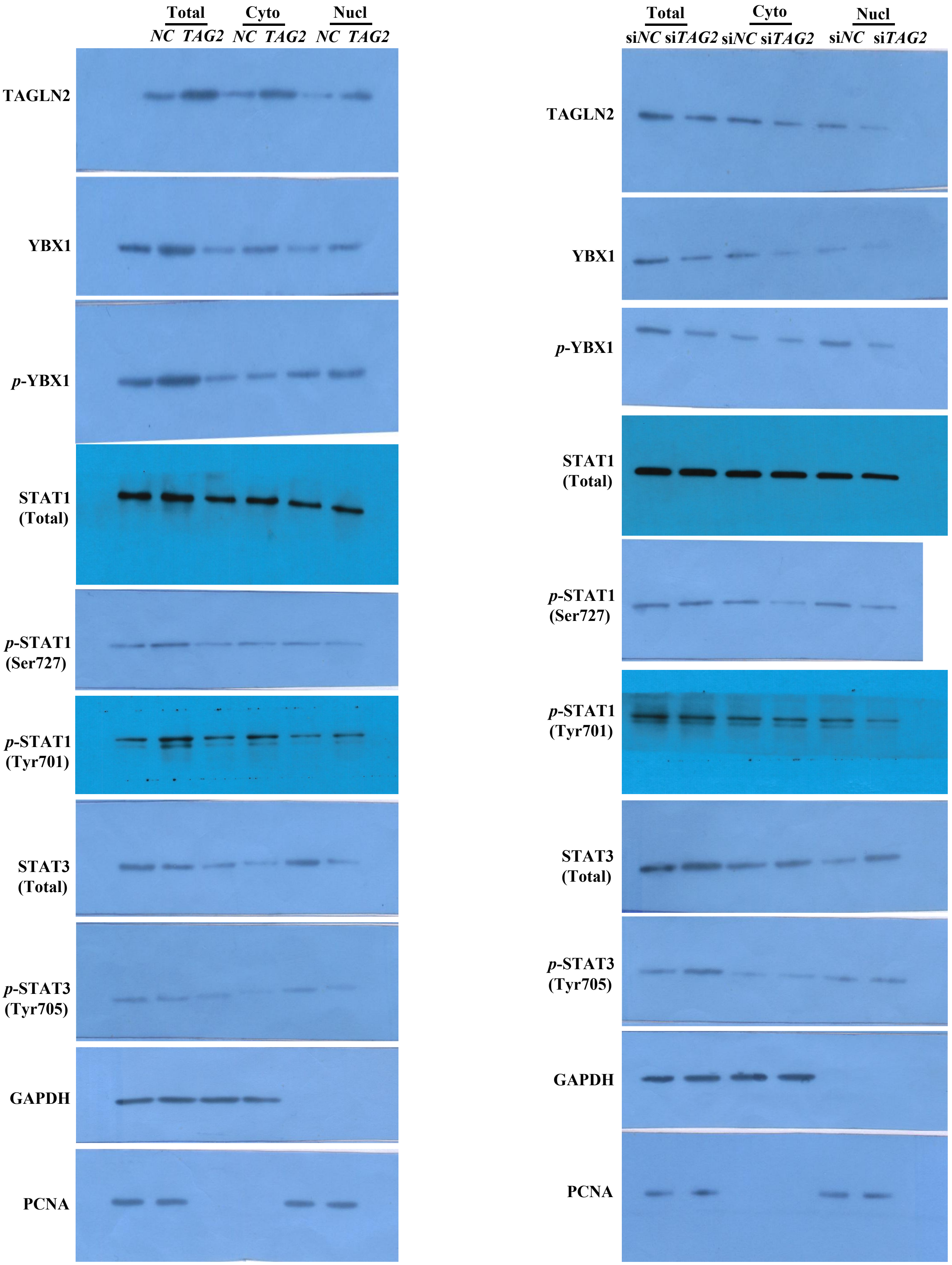

Total
Cyto
Nucl
NC
TAG2
NC
TAG2
NC
TAG2
TAGLN2
YBX1
p-YBX1
STAT1 (Total)
p-STAT1 (Ser727)
p-STAT1 (Tyr701)
STAT3 (Total)
p-STAT3 (Tyr705)
GAPDH
PCNA
Total
Cyto
Nucl
siNC
siTAG2
siNC
siTAG2
siNC
siTAG2
TAGLN2
YBX1
p-YBX1
STAT1 (Total)
p-STAT1 (Ser727)
p-STAT1 (Tyr701)
STAT3 (Total)
p-STAT3 (Tyr705)
GAPDH
PCNA

Figure 5F

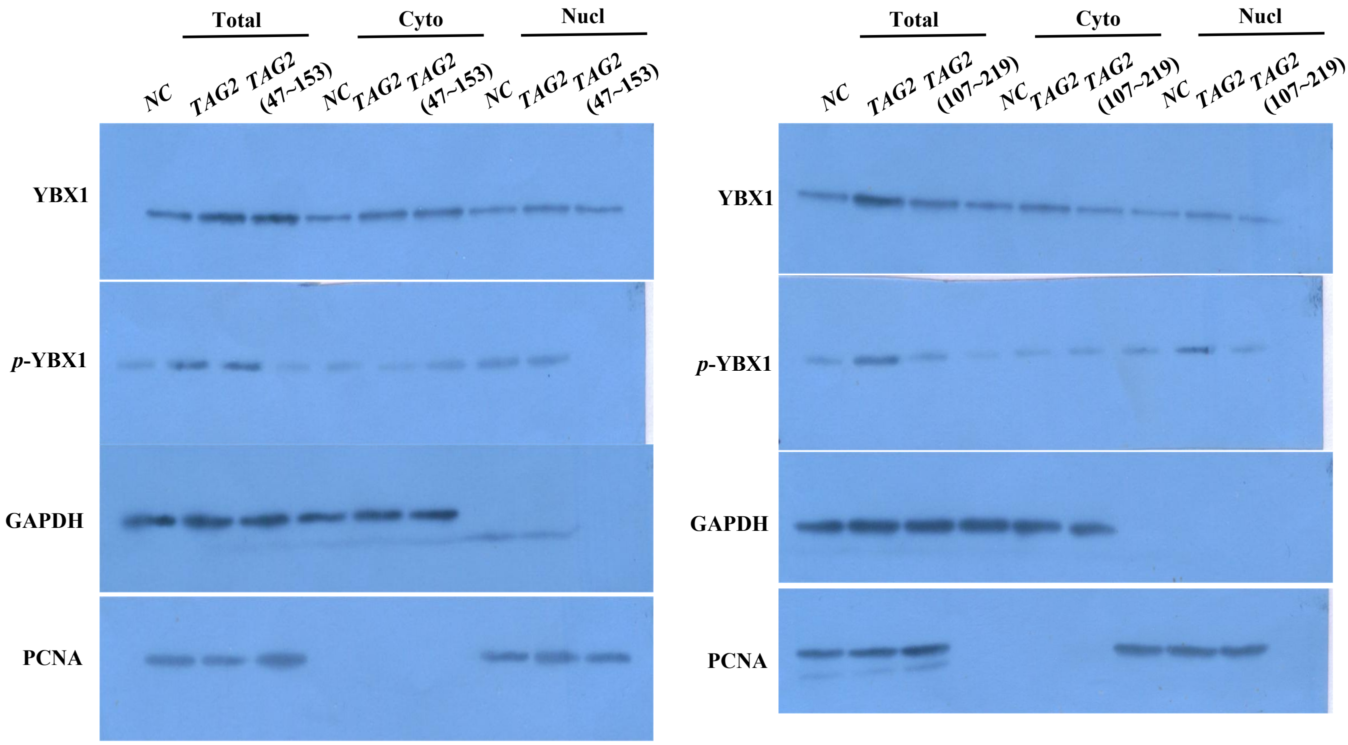

Supplementary Figure 1A

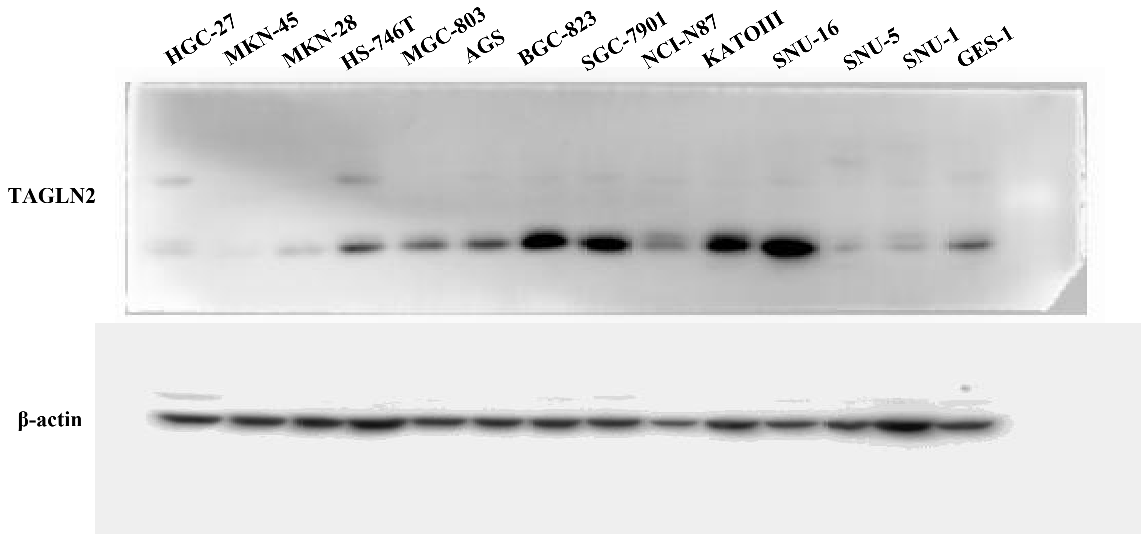

Supplementary Figure 1B

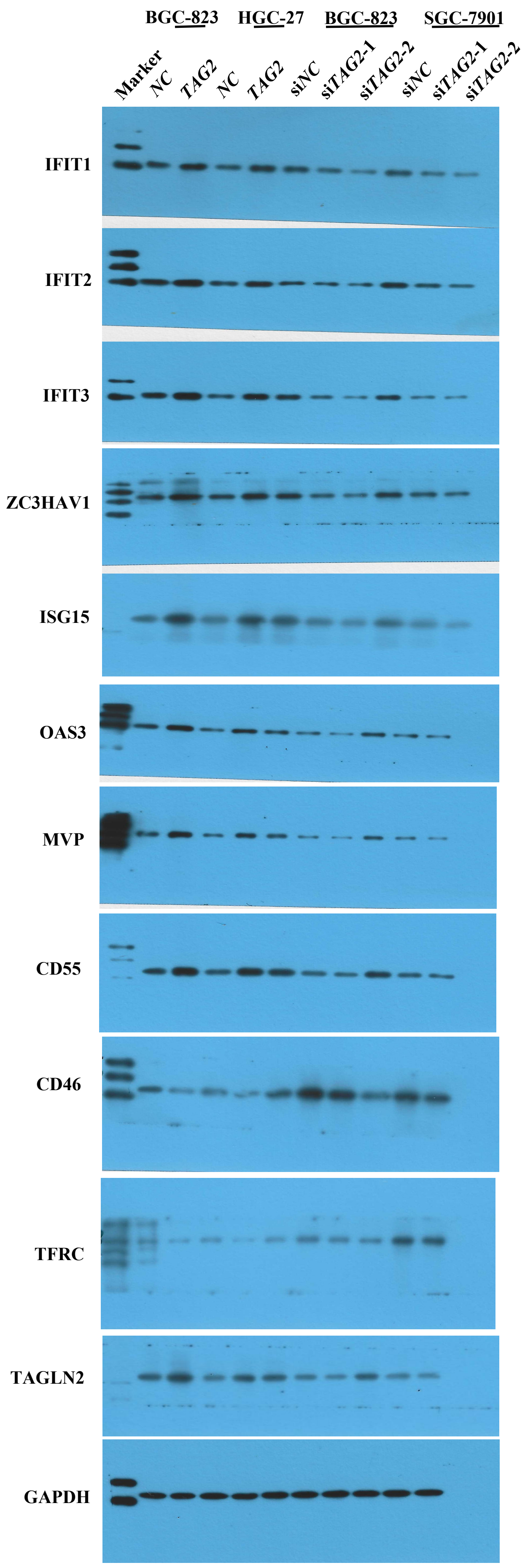

Supplementary Figure 2D

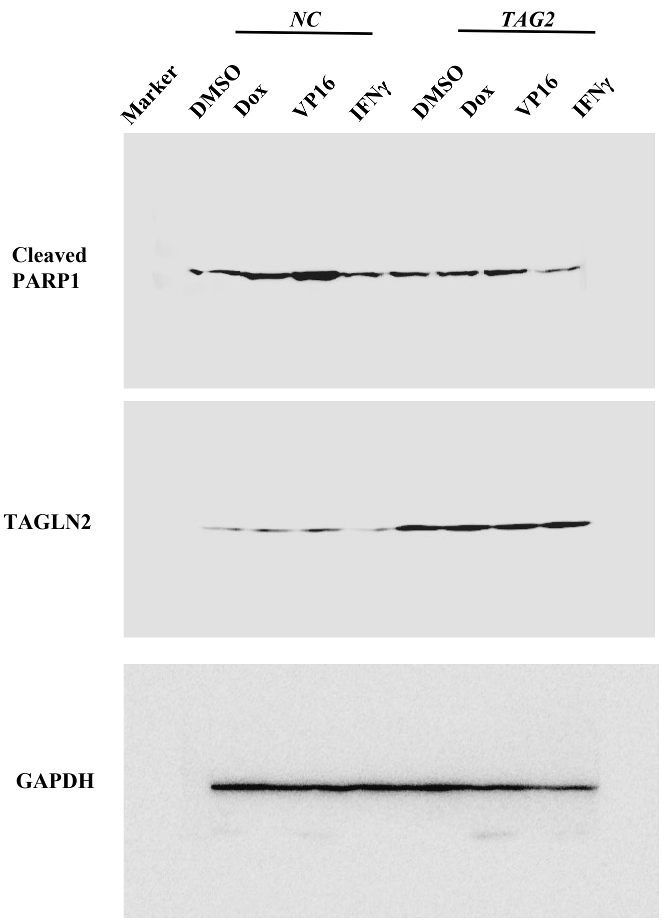

Supplement: Supplementary file 2 — Original data files [file 41419_2024_7000_MOESM2_ESM.pdf]
